# Implementation of monocular visual SLAM with ARCog-NET for aerial robot swarm indoor mapping

**DOI:** 10.1038/s41598-025-28618-x

**Published:** 2025-12-22

**Authors:** Gabryel Silva Ramos, Milena Faria Pinto, Fabio A. A. Andrade, Diego Barreto Haddad

**Affiliations:** 1 Control and Automation Laboratory (LACEA), Federal Center for Technological Education Celso Suckow da Fonseca (CEFET-RJ), Rio de Janeiro, 20271-110 Brazil; 2 Faculty of Technology, Natural Sciences and Maritime Sciences, University of South-Eastern Norway, 3184 Borre, Norway

**Keywords:** Engineering, Mathematics and computing

## Abstract

This paper presents a real-time distributed framework for Unmanned Aerial Vehicle (UAV) swarms operating in GPS-denied indoor environments, built on the Aerial Robot Cognitive Network Architecture (ARCog-NET) cognitive architecture and enhanced with monocular visual Simultaneous Localization and Mapping (SLAM). ARCog-NET employs a multi-layered Edge-Fog-Cloud (EFC) hierarchy that supports decentralized decision-making and adaptive coordination, including dynamic path optimization. Through a novel formulation, each UAV jointly estimates its own trajectory and contributes to a shared 3D reconstruction of the environment by exchanging matched visual landmarks across the network. The system dynamically adapts navigation paths in response to operational events using reinforcement learning guided by trajectory coverage metrics and historical decision weights. A full deployment with six DJI Ryze Tello UAVs was conducted in a controlled indoor lab environment, demonstrating autonomous swarm navigation and collaborative 3D mapping. Performance was evaluated through metrics such as trajectory error, point cloud fidelity, decision convergence, and knowledge reuse rate. Results confirm that the proposed method enables scalable, autonomous SLAM and planning capabilities in real-world UAV networks, highlighting the cognitive synergy between navigation and perception in distributed aerial robotics.

## Introduction

Studies on UAV (Unmanned Aerial Vehicle) swarm architectures typically address challenges of scalability, reliability, and energy efficiency^1^. Control models are commonly classified into centralized, decentralized, and hybrid schemes, each offering a trade-off between coordination simplicity and fault tolerance^2^. More recently, cognitive architectures have emerged as a promising alternative for dynamic environments, incorporating AI (Artificial Intelligence) and learning mechanisms to enable real-time decision-making and adaptability^3,4^. Systems such as Intelligent Vehicle Control Architecture (IVCA)^5^ and Aerostack^6^ provide modular coordination, while Selecky’s framework^7^ enables autonomous sensor-based operation.

In order to fill gaps in generic UAV swarm architectures to serve as a base for developing multi-aerial robots, proven both in simulation using real-world data and practical experimentation, therefore proposing solutions for the challenges in communication, control, and scalability pointed out in literature^1,2,4,8–11^, the Aerial Robot Cognitive Network Architecture (ARCog-NET)^3,12^ was proposed. It consists of a distributed cognitive framework designed to coordinate autonomous aerial robots operating collaboratively across Edge–Fog–Cloud (EFC) layers. It integrates real-time decision-making, visual perception, and reinforcement-based learning to support adaptive trajectory planning and cooperative task allocation and formation control among UAV swarms. By modeling knowledge reuse and hierarchical communication between agents, ARCog-NET enables resilient, context-aware behavior in dynamic and GPS-denied environments, bridging individual autonomy with collective intelligence.

The purpose of this paper is to use ARCog-NET’s flexible infrastructure, aligned with its built-in trajectory planning and formation control mechanisms based on cognitive learning and cooperative behavior, to build an efficient monocular SLAM (Simultaneous Localization and Mapping) module. This integration aims to enable multi-UAV collaborative mapping under GPS-denied conditions, where each agent contributes to a shared 3D reconstruction of the environment while autonomously adapting its trajectory based on visual feedback and collective knowledge. Through this approach, the framework extends beyond traditional distributed SLAM by embedding cognitive reasoning and hierarchical decision-making into the swarm, thus enhancing accuracy, scalability, and resilience in real-world aerial missions.

Monocular Simultaneous Localization and Mapping (SLAM) refers to the process by which a single camera estimates both the trajectory of a moving agent and the three-dimensional structure of its environment simultaneously. It represents one of the most fundamental perception capabilities in robotics, enabling autonomous agents to navigate and interact in unknown or GPS-denied environments without relying on external positioning systems. In robotic applications, particularly on lightweight aerial platforms where payload and power constraints limit the use of multiple sensors, monocular SLAM offers a cost-effective and computationally efficient alternative for localization and mapping. Its integration into swarm robotics further amplifies its relevance, allowing multiple UAVs to collectively reconstruct environments, share visual knowledge, and enhance spatial awareness through cooperative perception and distributed processing^13^.

Regarding cognition, in robotics, it is understood as the integration of perception, learning, and memory to achieve autonomy^14,15^. Techniques including neural networks, expert systems, evolutionary computing, and Reinforcement Learning (RL) have been employed to support adaptive decision-making in such systems^16^. Within ARCog-NET, RL is applied to update decision weights based on past performance, improving both efficiency and adaptability by reinforcing strategies that prove effective while discarding those that fail.

**Fig. 1:**
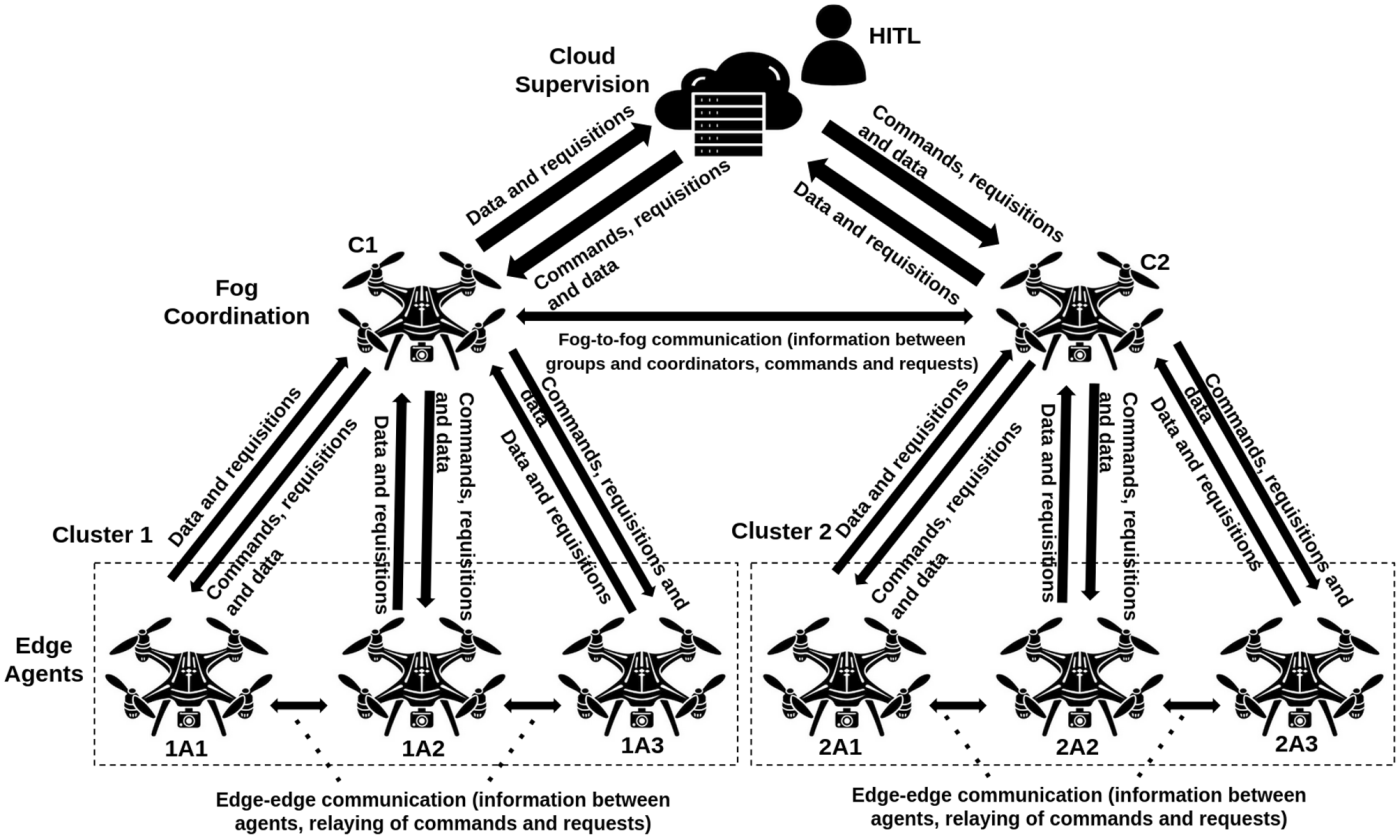
Structure of ARCog-NET with two clusters: two Fog-level coordinators and three Edge-level agents per group.

Several studies utilized ROS (Robot Operating System)^17^ and Gazebo simulation software^18^ for multi-UAV simulations, with integration of MAVROS^19^, PX4 firmware^20^, and the MAVLINK (Micro Air Vehicle Link) protocol^21^, enabling realistic testing. For instance^22^, explored fog-to-cloud distributed processing without cognitive learning integration. ARCog-NET incorporates RL to enhance task allocation, path planning, and formation control decisions. The architecture maintains a historical record of decision weights and coverage results, reinforcing effective strategies and discarding inefficient ones. This mechanism improves decisions such as whether to execute a task, follow a trajectory, or replicate the path of a neighboring UAV within a formation. It also supports HITL (Human-in-the-Loop) supervision and intervention from the cloud. Figure 1 presents a simplified representation of ARCog-NET and its communication flows, highlighting the hierarchical organization of two clusters and the corresponding communication flows. The architecture is comprised of cloud-level supervision with HITL, fog-level coordination through cluster coordinators, and edge-level operation by autonomous agents. Communication pathways are explicitly represented, including cloud-to-fog, fog-to-fog, and edge-to-edge exchanges, ensuring efficient command dissemination, data sharing, and inter-agent collaboration.

Cognition in ARCog-NET is modeled as an optimized task allocation problem, where the utility of each action must be maximized. Decision weights guide the system in choosing the best response to events or in generating new solutions. A task is defined as any action performed by the UAV in response to events (e.g., obstacle avoidance or covering the failure of a neighbor). The allocation of these tasks is modeled as a decision-making process in which the system evaluates the potential utility of alternative actions. Decision weights are then applied to balance multiple criteria, such as mission objectives, safety, and energy consumption, guiding the UAV to select the most suitable response. This process is formulated as an optimization problem to ensure that tasks are allocated efficiently, thereby improving overall system performance and robustness. If a UAV cannot optimally allocate the task, it may escalate the request to a higher layer or collaborate with peers at the same level. This mechanism allows layers to function both independently and cohesively within the cognitive architecture.

Two UAV navigation-specific tasks execute distinct cognitive routines: trajectory planning and formation control. The first computes paths or delegates the solution to upper layers, learning from each execution. The second is triggered when UAVs must fly in proximity, either following similar paths or executing HITL-defined missions. These two routines are interdependent, i.e., effective formation control requires reliable path planning, while path adjustments may be constrained by formation constraints. The cognitive framework was designed this way as one mode complements the other, resulting in effective group flight with less processing (for example, if a UAV group has similar trajectories, the path planning will be triggered to just one of them, and the other will enter in formation control). Thus, ARCog-NET implements three cascading cognitive algorithms: one for general task allocation and two for flight-specific cognitive subroutines. This study is mainly based on path planning-related and navigation tasks^3^.

Regarding distributed computing applications on robotic swarms, Tahir’s comprehensive survey on edge and fog robotics highlights the emerging importance of fog robotics in multi-robot systems, providing low-latency offloading, local autonomy, and scalable coordination, foundational concepts that align with the design philosophy of ARCog-NET^23^. In this scenario, ARCog-NET advances over survey-level architectures by demonstrating real UAV swarm deployment with integrated SLAM, cognitive navigation, and experimental validation.

Recent years have witnessed substantial advances in distributed SLAM (Simultaneous Location and Mapping) for UAV swarms. For example, Friess et al. proposed a fully onboard SLAM framework for nano-UAVs, capable of generating coherent grid maps using only low-power microcontrollers and limited sensing capabilities and achieving mapping accuracies of around 12 cm with minimal infrastructure dependence^24^. This work demonstrates impressive scalability and autonomy. However, it lacks higher-level cognitive coordination and dynamic trajectory optimization, which ARCog-NET provides.

Xu et al. introduced D$\phantom{0}^2$SLAM^25^, a decentralized and distributed visual-inertial SLAM system designed for aerial swarms. It supports both near-field relative estimation and far-field global consistency, with robustness to network delays and scalability improvements via distributed optimization. While D$\phantom{0}^2$SLAM focuses on SLAM robustness, ARCog-NET enriches this by integrating distributed cognitive decision-making, trajectory adaptation, and reinforcement learning.

In the LiDAR domain, Zhong et al. presented Distributed Collaborative LiDAR SLAM (DCL-SLAM)^26^, a collaborative framework for robotic swarms that enables distributed loop closure and pose graph optimization using lightweight descriptors over an ad-hoc network. Their architecture emphasizes communication efficiency and inter-robot consistency, yet doesn’t address higher-level navigation and mapping synergy across ARCog-NET’s Edge-Fog-Cloud layers.

Moreover, task offloading strategies have gained traction in recent literature. Aldossary et al. formulated a Mixed-Integer Linear Programming (MILP)-based collaborative offloading strategy, showing significant reductions in service latency (up to 59%) and energy consumption (up to 12%) via optimized allocation across UAVs, fog, and cloud nodes^27^. Similarly, Hu et al. proposed a swarm-enabled mobile edge computing system combining task offloading and data compression, using deep reinforcement learning (PER-DDPG) to minimize latency and energy costs by approximately 32%^28^. These works align with ARCog-NET’s multi-layer cognition, though ARCog-NET uniquely integrates SLAM-derived mapping and trajectory adaptation with cognitive reinforcement.

Novel studies have further advanced distributed and collaborative SLAM frameworks through cloud-edge integration and multimodal feature fusion. Liu et al^29^. proposed an edge-assisted multi-robot visual-inertial SLAM approach that enhances real-time performance by reducing communication overhead via feature compression, demonstrating efficient coordination between cloud, edge, and robotic agents. Complementarily, Liu et al^30^. introduced CPL-SLAM, a centralized collaborative visual-inertial system that combines point and line features to improve localization accuracy and robustness in multi-robot scenarios. These contributions highlight the growing emphasis on hierarchical computation and cross-agent data sharing-principles that align closely with the cognitive and multi-layered decision-making design of ARCog-NET.

Recent advances have explored deep reinforcement learning and path planning for autonomous robotic navigation in unstructured environments. Zhang et al. (2025) proposed a LiDAR-based exploration method for mobile robots that integrates deep reinforcement learning to enhance adaptability and real-time decision-making in unknown spaces^31^. Similarly, Zhang et al. (2024) introduced the E-Planner algorithm, which leverages visibility graphs to optimize path planning efficiency and obstacle avoidance in dynamic scenarios^32^. Both studies highlighted the growing trend of coupling learning-based decision strategies with classical mapping and planning frameworks, an approach conceptually aligned with ARCog-NET’s cognitive reinforcement mechanisms and hierarchical coordination across the Edge–Fog–Cloud layers.

To contextualize the contributions of ARCog-NET, it is compared with recent distributed SLAM and cognitive UAV frameworks. While prior approaches have emphasized onboard resource constraints, visual-inertial consistency, or communication-efficient collaboration, ARCog-NET uniquely integrates multi-layer cognitive decision-making, adaptive trajectory optimization, and 3D mapping through swarm intelligence. Table 1 summarizes the key parameters considered in each study.

**Table 1:** Feature comparison among ARCog-NET and recent UAV swarm and distributed SLAM frameworks.

**Study**	**Mapping**	**Odometry**	**Cognitive** **Planning**	**Trajectory** **Adaptation**	**Communication** **Model**	**Multi-layer** **Processing**
Friess et al.^24^	$\checkmark$	$\checkmark$	–	–	Local-only	–
Xu et al.^25^	$\checkmark$	$\checkmark$	–	–	Decentralized	–
Zhong et al.^26^	$\checkmark$	$\checkmark$	–	–	Ad-hoc	–
Aldossary et al.^27^	–	–	$\checkmark$	–	Fog-Cloud	$\checkmark$
Hu et al.^28^	–	–	$\checkmark$	$\checkmark$	Fog-Cloud	$\checkmark$
Zhang et al.^32^	–	–	$\checkmark$	$\checkmark$	Local-only	–
Zhang et al.^31^	$\checkmark$	–	$\checkmark$	$\checkmark$	Local-only	–
Liu et al.^29^	$\checkmark$	$\checkmark$	–	–	Edge-Cloud	$\checkmark$
Liu et al.^30^	$\checkmark$	$\checkmark$	–	–	Centralized	–
**ARCog-NET**	$\checkmark$	$\checkmark$	$\checkmark$	$\checkmark$	Edge-Fog-Cloud	$\checkmark$

Overall, while existing works have substantially advanced distributed SLAM and multi-layer offloading, ARCog-NET combines cognitive trajectory decision-making, SLAM-based mapping, and hierarchical Edge-Fog-Cloud architecture in a real-world multi-UAV deployment context.

## Methods and modelling

The ARCog-NET architecture assumes that UAVs operate in a 3D space $A \in \mathbb {R}^3$. This spatial domain physically represents the real operational volume of the swarm, where each coordinate (*x*, *y*, *z*) corresponds to a measurable position in meters relative to the laboratory environment. This distributed system consists of nodes structured into two operational levels: edge agents $e=\{e_1, e_2,..., e_m\}$ and fog coordinators $f=\{f_1, f_2,..., f_n\}$, where $e_m$ and $f_n$ define the maximum number of active units per layer. In physical terms, edge agents correspond to individual UAVs performing perception and navigation tasks, while fog coordinators act as higher-level processors integrating data from nearby UAVs to manage coordination and local map fusion. At deployment ($t_i$), UAVs, their group assignments, and fog coordination roles are configured via Human-In-The-Loop (HITL) supervision at the cloud layer^3,12^. The HITL initialization physically represents the preflight configuration and safety validation process before takeoff, equivalent to defining initial mission parameters and ensuring communication readiness.

While flying, each UAV $i$ at layer $L$ (Note that when the notation $^{L_i}$ or $^{L'_j}$ appears before the variable symbol, it is related to computer vision. On the other hand, if these notations appear after the variable symbol, they are related to navigation.) (Fog or Edge) gather a sequence of monocular camera frames captured from time $t = t_i$ to $t = t_n$, denoted as: $^{L_i}I = \{^{L_i}I_0, ^{L_i}I_1, ^{L_i}I_2, \ldots , ^{L_i}I_{t_n}\}$. Here, each $^{L_i}I_t$ corresponds to a real RGB image frame captured by the onboard camera, representing the projection of the surrounding 3D environment onto a 2D plane at time *t*. The supervisor may also set initial waypoints and define deviations due to operational demands. UAVs initially follow predetermined flight paths ($t=0$), either generated autonomously or predefined, but these routes can be adjusted at any moment $t> 0$ to satisfy dynamic requirements. The initial set of waypoints for UAV *i* in a given layer $L \in \{e, f\}$ is $n^{L_i}$, while the dynamically updated number of waypoints at time *t* is $n^{L_i}_p(t)$, computed as:

1 $$\begin{aligned} n^{L_i}_p(t) = \sum _{\iota =1}^M \sum _{\gamma =1}^N \sum _{k=1}^O P_{L_i}^\kappa (x_\iota , y_\gamma , z_k), \end{aligned}$$

being *M*, *N* and *O* the maximum number of (*x*, *y*, *z*) trajectory coordinates. Physically, this triple summation represents the discrete sampling of the UAV trajectory through the 3D workspace, where each $P_{L_i}^\kappa (x_\iota , y_\gamma , z_k)$ corresponds to an attainable spatial waypoint determined by navigation commands and sensor feedback. This expression reflects the number of waypoints involved in the mission at a given time *t*, already reached or to be navigated, adapting to events that trigger navigation tasks ($T_\text {nav}$). $T_\text {nav}$ thus corresponds to any detected operational stimulus-such as obstacle proximity or communication loss-that requires a physical redirection of the UAV path. Each term $P_{L_i}^\kappa (x_\iota , y_\gamma , z_k)$ indicates a navigation point on trajectory $\varrho _{L_i}$. In addition to the navigation points, each UAV will carry a local map of the environment, composed of 3D landmarks $^{L_i}M = \{^{L_i}p_1, ^{L_i}p_2, ^{L_i}p_3, \ldots , ^{L_i}p_t\}$ where each $p_t$ is a 3D point in the environment reconstructed from visual data. In physical terms, these landmarks correspond to identifiable features-such as walls, corners, or obstacles-whose 3D coordinates are reconstructed from monocular vision through depth inference and triangulation. The objective of ARCog-NET Monocular SLAM is to cognitively align the traditional navigation task points $n^{L_i}_p(t)$ with the 3D map $^{L_i}M$, so the traditional navigation algorithm of ARCog-NET can both feed the visual odometry and mapping and use mapping information to improve itself in a virtuous cycle. This coupling represents a feedback process between physical motion (control commands and inertial movement) and perceptual information (camera observations), ensuring that UAV decisions are grounded in measurable environmental geometry.

As UAV flies, the objective is to maximize the joint posterior probability of the camera (or the UAV itself) trajectory $n^{L_i}_p(t)$ and the map $^{L_i}M$ given the visual observations $^{L_i}I$, denoted $P(n^{L_i}_p(t), ^{L_i}M \mid ^{L_i}I)$. This probabilistic formulation has a physical interpretation as an optimization over all possible flight paths and 3D structures that best explain the visual evidence gathered by the UAV’s camera. According to Bayes’ theorem, this posterior can be factorized as:

2 $$\begin{aligned} P(n^{L_i}_p(t), ^{L_i}M \mid ^{L_i}I) = P(^{L_i}M \mid n^{L_i}_p(t), ^{L_i}I) \cdot P(n^{L_i}_p(t) \mid ^{L_i}I), \end{aligned}$$

where $P(^{L_i}M \mid n^{L_i}_p(t), ^{L_i}I)$ represents the posterior probability of the local environment map $^{L_i}M$ given the estimated UAV trajectory $n^{L_i}_p(t)$ and its image observations $^{L_i}I$ and $P(n^{L_i}_p(t) \mid ^{L_i}I)$ is the posterior probability of the camera trajectory $n^{L_i}_p(t)$ conditioned only on the sequence of image observations $^{L_i}I$. Physically, the first term models how reliable the reconstructed environment is given the flight path, while the second quantifies the consistency of UAV motion estimation relative to sensor measurements. Thus, Monocular Visual SLAM in ARCog-NET is naturally decomposed into two interrelated sub-problems: odometry and mapping, which together enable the simultaneous estimation of the agent’s motion and the 3D structure of the observed environment.

To gain a clearer understanding of how the local and global 3D maps are formed alongside navigation, it is first necessary to model the image formation of each UAV $L_i$. In the proposed test using the vision system of the Tello UAV, spatial relationships are defined with respect to coordinate frames. The world coordinate frame is a fixed, global reference frame used to describe the absolute position and orientation of objects in a 3D environment. Its origin can be arbitrarily chosen; in this work, it is defined as the lower-left corner of the laboratory. This means that all UAV poses and reconstructed points are physically measured relative to this laboratory-fixed coordinate system, ensuring consistency across agents during swarm mapping.

On the other hand, the camera coordinate frame is the local frame centered at the camera’s optical center. It represents the position of objects relative to the camera. The transformation between the world and camera of the UAV $L_i$ frames is defined by the camera’s extrinsic parameters, represented as $[^{L_i}\mathscr {R}\mid ^{L_i}\mathscr {T}]$, where $^{L_i}\mathscr {R} \in \mathbb {R}^{3 \times 3}$ is the rotation matrix and $^{L_i}\mathscr {T} \in \mathbb {R}^{3 \times 1}$ is the translation vector. In physical terms, $^{L_i}\mathscr {R}$ captures the UAV’s orientation in 3D space (roll, pitch, yaw), while $^{L_i}\mathscr {T}$ represents its position vector in meters relative to the global origin. This matrix allows conversion between world and camera coordinates, enabling localization, mapping, and visual odometry.

**Fig. 2:**
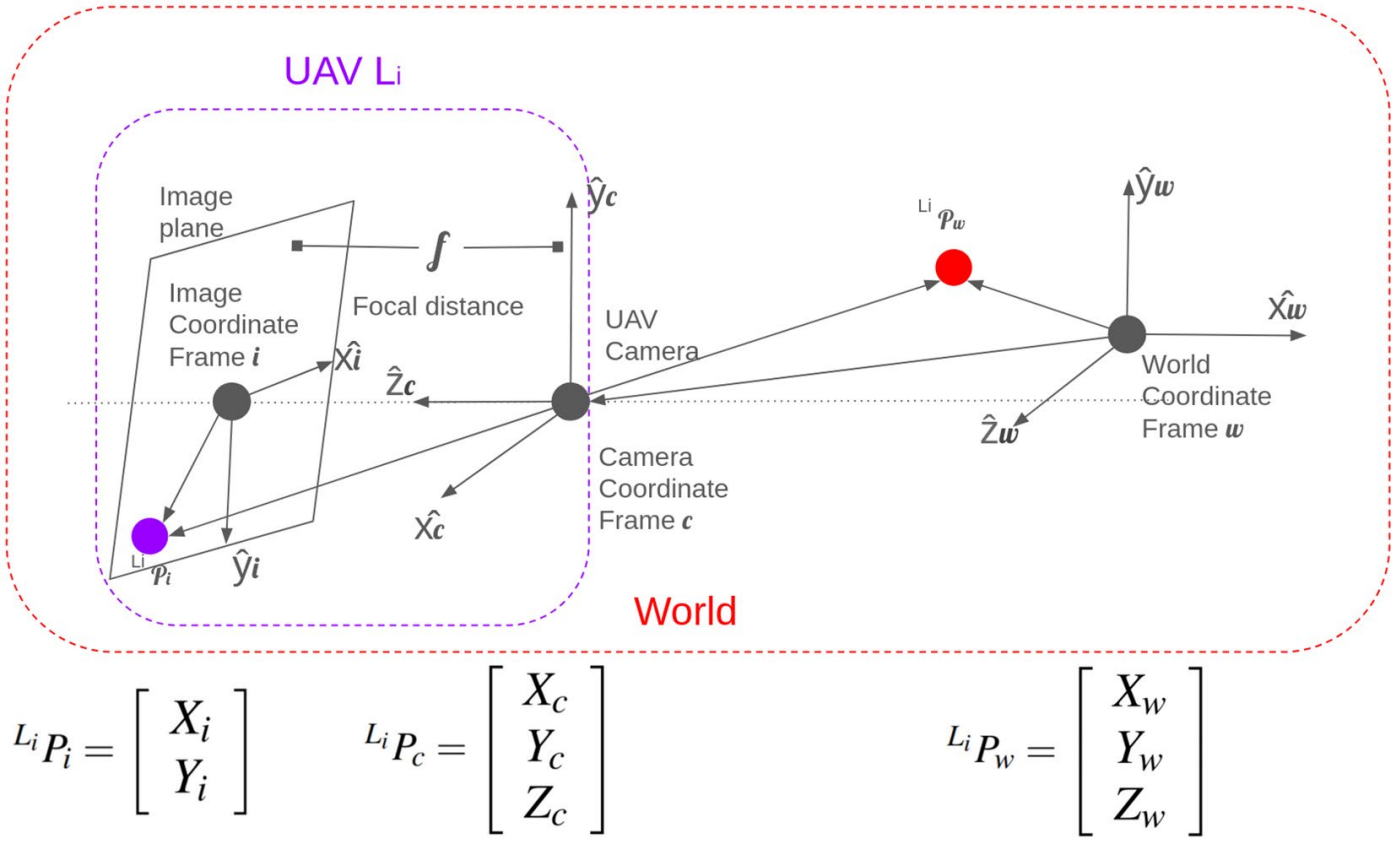
World 3D point in monocular 2D image.

In the same way, the image coordinate frame is a 2D reference system that represents the position of points on the image plane. It is related to the camera’s 3D coordinate frame through the intrinsic camera parameters, also known as the intrinsic matrix of UAV $L_i$ $^{L_i}\mathscr {K}$. This matrix physically encodes the camera focal length and optical center, determining how 3D world points are projected onto pixel coordinates. To project a 3D point from the world onto the image, a two-step transformation is applied, i.e., first the extrinsic matrix $[^{L_i}\mathscr {R} \mid ^{L_i}\mathscr {T}]$ transforms the point from the world coordinate frame to the camera coordinate frame (3D to 3D) and then the intrinsic matrix $^{L_i}\mathscr {K}$ transforms the point from the camera frame to the image plane (3D to 2D). The complete projection process combines both matrices, enabling the camera to represent 3D world geometry as 2D image data, as presented in Figure 2. Physically, this transformation pipeline reproduces the imaging process by which a real-world object at position (*x*, *y*, *z*) appears as pixel coordinates (*u*, *v*) on the UAV’s camera sensor, closing the loop between physical environment and digital perception.

When a UAV camera captures an image, the 3D coordinates of a point in the world frame are first transformed into the camera coordinate frame using the extrinsic matrix, which includes both rotation and translation components, represented by:

3 $$\begin{aligned} ^{L_i}P_c = [^{L_i}\mathscr {R} \mid ^{L_i}\mathscr {T}] \, ^{L_i}P_w = ^{L_i}\mathscr {R} \, ^{L_i}P_w + ^{L_i}\mathscr {T}. \end{aligned}$$

Physically, this equation represents the transformation of a real point $^{L_i}P_w$ in the 3D world (expressed in meters within a fixed laboratory reference frame) into its equivalent coordinates $^{L_i}P_c$ relative to the UAV camera. The rotation matrix $^{L_i}\mathscr {R}$ describes the UAV’s orientation in space (roll, pitch, and yaw), while the translation vector $^{L_i}\mathscr {T}$ indicates its position offset in meters with respect to the global origin. This operation defines how the UAV “sees” the world from its current pose.

Once in the camera frame, the point is projected onto the 2D image plane using the intrinsic matrix $^{L_i}\mathscr {K}$, which encodes the UAV camera’s internal parameters $^{L_i}P_i = ^{L_i}\mathscr {K} \, ^{L_i}P_c$.

In physical terms, this second transformation models the imaging process through the UAV’s camera lens, where the 3D coordinates are converted into 2D pixel positions (*u*, *v*). The intrinsic matrix $^{L_i}\mathscr {K}$ contains the focal length and principal point, defining how light rays from real-world positions are projected onto the image sensor, producing measurable pixel displacements.

While this suffices to explain how a 3D world point is represented in a local UAV image plane, the reconstruction of the 3D environment as a point cloud and the flight planning through this environment still need additional steps. This reconstruction physically corresponds to estimating the actual geometry of the environment-walls, obstacles, and landmarks-from the multiple overlapping 2D images captured during flight. To better understand how this is done, it is important to notice that ARCog-NET navigation consists in generating 3D trajectory points for the UAV to fly stochastic exploration, and the trajectory is improved by reinforcement learning as both the subject and the swarm learns about mission objectives and the environment they’re operating. In practical terms, this means that the UAV continuously refines its motion path in real-world space based on sensor feedback and cooperative learning, gradually improving coverage and safety. Whenever an event is detected during flight, the system will recalculate the trajectories based on self-knowledge or collective knowledge decisions. An event may have various types, from a loss of a communication link to an obstacle detection. The trajectory point increment due to event-driven updates is defined in Equation (4).

4 $$\begin{aligned} \Delta P^\kappa (t - t_i) = {\left\{ \begin{array}{ll} \delta ^\kappa _p, & \text { if } ||P_{L_i}^\kappa (t)_\text {new}|| - ||P_{L_i}^\kappa (t)_\text {old}|| \ge 0, \\ - \delta ^\kappa _p, & \text { if } ||P_{L_i}^\kappa (t)_\text {new}|| - ||P_{L_i}^\kappa (t)_\text {old}|| < 0, \end{array}\right. } \end{aligned}$$

Equation (4) physically expresses the displacement $\Delta P^\kappa (t-t_i)$ of a UAV between two instants, corresponding to how much its real position in 3D space changed after an event. The scalar $\delta ^\kappa _p$ quantifies this spatial correction in meters, and the $\ell _2$-norm measures the Euclidean distance traveled. A positive increment indicates forward progression in the mission area, while a negative one represents retreat or obstacle avoidance maneuvers.

where $\delta ^\kappa _p$ captures the magnitude of change in the point’s position due to the event, and $\Vert \cdot \Vert$ denotes the $\ell _2$-norm. Each UAV’s current objective at time *t* is denoted $P^{L_i}_{\text {obj}_j}(t) \in \mathbb {R}^3$, and its trajectory is dynamically adapted based on mission events. The motion of such an objective is defined by:

5 $$\begin{aligned} {\left\{ \begin{array}{ll} \theta ^{L_i}_{\text {obj}_j}(t) = \arctan \left( \frac{{P^{L_i}_{\text {obj}_j}}[y](t_i) - {P^{L_i}_{\text {obj}_j}}[y](t)}{{P^{L_i}_{\text {obj}_j}}[x](t_i) - {P^{L_i}_{\text {obj}_j}}[x](t)}\right) \\ V^{L_i}_{\text {obj}_j}(t) = \frac{||P^{L_i}_{\text {obj}_j}(t)-P^{L_i}_{\text {obj}_j}(t_i)||}{t-t_i}\\ P^{L_i}_{\text {obj}_j}[x](t_{i+1}) = P^{L_i}_{\text {obj}_j}[x](t) + V^{L_i}_{\text {obj}_j}(t) \cdot \cos \theta ^{L_i}_{\text {obj}_j}(t) \cdot \Delta t \\ P^{L_i}_{\text {obj}_j}[y](t_{i+1}) = P^{L_i}_{\text {obj}_j}[y](t) + V^{L_i}_{\text {obj}_j}(t) \cdot \sin \theta ^{L_i}_{\text {obj}_j}(t) \cdot \Delta t, \end{array}\right. } \end{aligned}$$

Equation (5) represents the physical kinematic model governing UAV motion toward a mission objective. The heading $\theta ^{L_i}_{\text {obj}_j}(t)$ corresponds to the real orientation angle in radians, derived from the relative displacement between past and current positions. The velocity $V^{L_i}_{\text {obj}_j}(t)$ represents the UAV’s true linear speed (m/s), calculated by dividing the traveled distance by elapsed time. The last two expressions describe how these quantities update the UAV’s Cartesian coordinates (*x*, *y*) over a discrete time step $\Delta t$, modeling continuous flight through sequential motion increments.

In this equation, where $\theta ^{L_i}_{obj_j}(t)$ denotes the heading angle of UAV *i* in layer *L* with respect to objective *j* at time *t*, obtained from the relative displacement along the *x* and *y* axes, while $v^{L_i}_{obj_j}(t)$ indicates its velocity magnitude. The UAVs maintain a fixed operational altitude, adapting it only within a safe range during navigation, and follow a vectorial motion model:

6 $$\begin{aligned} {\left\{ \begin{array}{ll} \theta _{L_i}(t) = \arctan \left( \frac{P_{L_i}[y](t_i) - P_{L_i}[y](t)}{P_{L_i}[x](t_i) - P_{L_i}[x](t)}\right) \\ V_{L_i}(t) = \frac{||P_{L_i}(t)-P_{L_i}(t_i)||}{t-t_i}\\ P_{L_i}[x](t_{i+1}) = P_{L_i}[x](t) + V_{L_i}(t) \cdot \cos \theta _{L_i}(t) \cdot \Delta t\\ P_{L_i}[y](t_{i+1}) = P_{L_i}[y](t) + V_{L_i}(t) \cdot \sin \theta _{L_i}(t) \cdot \Delta t, \end{array}\right. } \end{aligned}$$

Equation (6) expresses the real planar motion of a UAV during navigation. The heading $\theta _{L_i}(t)$ gives the instantaneous flight direction, while $V_{L_i}(t)$ represents its measured translational velocity derived from displacement. The coordinate updates for $P_{L_i}[x]$ and $P_{L_i}[y]$ physically describe how the UAV’s position changes on the horizontal plane per time step $\Delta t$, corresponding to actual odometric movement tracked via onboard sensors or vision-based localization.

The trajectory coverage $C_{L_i}(t)$, in its turn, is a critical parameter of the ARCog-NET model, and it is defined as the set of points UAV *i* successfully navigated between *t* and $t+\tau$. Physically, $C_{L_i}(t)$ represents the portion of the environment (area or volume in $\textrm{m}^2$ or $\textrm{m}^3$) that has actually been visited, scanned, or mapped by UAV *i* over a given time interval, and therefore corresponds to effective spatial exploration progress. The total trajectory coverage is calculated by:

7 $$\begin{aligned} {\left\{ \begin{array}{ll} C^\kappa _{L_i}(t-t_i) = \Delta P_\kappa (t-t_i)\\ C^{\text {total}}_{L_i} = \frac{1}{n^{L_i}_p}\sum _{k=0}^{n^{L_i}_p}C^\kappa _{L_i}(t-t_i) \end{array}\right. }, \end{aligned}$$

In Equation (7), $C^\kappa _{L_i}(t-t_i)$ denotes the incremental contribution of a trajectory segment to the explored region, which is directly related to the physical path increment $\Delta P_\kappa (t-t_i)$ that the UAV actually flew in 3D space. The term $C^{\text {total}}_{L_i}$ is the accumulated fraction of mission waypoints covered by UAV *i*, normalized by $n^{L_i}_p$, and can be interpreted as a coverage efficiency score: values closer to 1 indicate that most intended locations have been physically reached and inspected.

and the value $C^{\text {total}}_{L_i}$ then defines the performance reward for trajectory adaptation $C_{L'_jL_i}^{PP}(t) = C^{\text {total}}_{L_i}$. Operationally, this means that higher physical coverage of the environment translates into higher reward during decision-making, incentivizing UAV behaviors that expand explored volume, avoid redundant revisits, and close blind spots in the map.

Given that the ARCog-NET framework enables each connected UAV to access the knowledge base of peers within its cluster (edge level), across clusters (fog level), or throughout the entire system (cloud level), the decision-making capability of the swarm is significantly enhanced. In physical terms, this hierarchical access corresponds to real wireless exchange of navigation history, mapped landmarks, and collision experiences between UAVs and coordinators, allowing one agent to benefit from another agent’s prior flight without having to re-fly the same region. This hierarchical access model improves the network’s capacity to find optimal solutions and further supports HITL intervention when autonomous reasoning fails to yield a safe or viable output. The trajectory coverage history of UAV *i* in layer *L* at a time instant *t* is stored in the following matrix:

8 $$\begin{aligned} H_{L_i}(t) = \left[ {\begin{array}{ccc} C^{L'_1}_{L_1}(t) & \cdots & C^{L'_1}_{L_n}(t)\\ \vdots & \ddots & \vdots \\ C^{L'_m}_{L_1}(t) & \cdots & C^{L'_m}_{L_n}(t) \end{array} } \right] . \end{aligned}$$

The matrix $H_{L_i}(t)$ therefore encodes, for UAV *i*, which parts of the environment were physically explored by which assisting node $L'_j$ (peer edge node, fog coordinator, or higher layer) at time *t*. Each entry $C^{L'_j}_{L_k}(t)$ can be interpreted as the measured contribution of node $L'_j$ to the spatial coverage achieved by node $L_k$, e.g., how much of the currently relevant area was already mapped or cleared of obstacles thanks to shared information rather than a new flight.

At the occurrence of an operational event, the choice to adjust the trajectory of UAV *i* in layer *L*, as influenced by node *j* in layer $L'$, will favor the option that results in the highest coverage performance. In other words, when something happens in the environment (for example, a blocked corridor, unexpected personnel movement, or link degradation), UAV *i* will prefer guidance from whichever node *j* has historically led to better physical area coverage and safer motion in comparable conditions. Notably, a UAV only retains trajectory coverage values for computations performed either by itself or by superior layers with which it has direct communication. This design ensures reliability and prevents the propagation of uncertain or outdated information. Otherwise, the coverage term is zero, i.e., $C^{L'_j}_{L_i}(t)=0$. This implies that the only knowledge propagated through $H_{L_i}(t)$ is grounded in physically executed motion or verified supervision, which prevents contamination by speculative or stale plans. The cumulative memory of a UAV’s decision-making process is organized into its knowledge base, described by $\kappa _{L_i} = \{ H_{L_i}(t_0), H_{L_i}(t_1), \cdots , H_{L_i}(t_n) \}$, where $H_{L_i}(t)$ denotes the trajectory coverage history. Thus, $\kappa _{L_i}$ can be interpreted as the long-term record of “where we have actually been and who helped us get there,” which persists across the mission and can be reused when similar situations reappear.

This knowledge base can initially be empty, with the UAVs acting solely on local decisions or frequently deferring to human operators during the early mission stages. Over time, as more operational data is acquired, reliance on HITL decreases, and the decision-making becomes more autonomous and informed. Each time a trajectory decision must be evaluated, a corresponding decision weight matrix is generated to accompany the coverage history:

9 $$\begin{aligned} W_{L_i}(t) = \left[ {\begin{array}{ccc} w^{L'_1}_{L_1}(t) & \cdots & w^{L'_1}_{L_n}(t)\\ \vdots & \ddots & \vdots \\ w^{L'_m}_{L_1}(t) & \cdots & w^{L'_m}_{L_n}(t) \end{array} } \right] . \end{aligned}$$

The matrix $W_{L_i}(t)$ attaches a physical meaning of trust or influence to each collaborating node. Each element $w^{L'_j}_{L_i}(t)$ represents how reliable node $L'_j$ has proven to be, in practice, when suggesting motion for UAV *i* in layer *L*. High weights correspond to guidance that previously led to safe flight, efficient coverage, low collision risk, or fast completion of tasks in real space. Lower weights reflect advice that produced inefficient detours, communication delays, or near-collision behavior.

In this matrix, $w^{L'_j}_{L_i}(t)$ represents the decision quality (or influence weight) associated with a trajectory recommendation for UAV *i* in layer *L*, as suggested by node *j* from layer $L'$. Each entry in $H_{L_i}(t)$ is tied to a corresponding weight in $W_{L_i}(t)$, offering a paired perspective on coverage quality and decision effectiveness. Together, $H_{L_i}(t)$ and $W_{L_i}(t)$ mean that ARCog-NET does not only record *what physical areas were explored*, but also *whose guidance produced that exploration most effectively in the real environment*. The combination of these elements enables the swarm to refer back to past experiences for context-aware reasoning. The long-term memory of these decision weights, representing UAV *i*’s history of learned effectiveness across various trajectory recommendations, is formalized as $\omega _{L_i} = \{ W_{L_i}(t_0), W_{L_i}(t_1), \cdots , W_{L_i}(t_n) \}$. $\omega _{L_i}$ therefore encodes, over time, the physically validated cooperation patterns that worked best for UAV *i*, allowing it to bias future navigation toward strategies that have already been proven safe and productive in similar indoor conditions.

Together, this structure of historical coverage and weighted reasoning allows UAVs in ARCog-NET to make informed decisions that not only support individual navigation goals but also contribute to cooperative environment mapping and overall network intelligence. In practice, this means that UAVs do not plan trajectories in isolation: they inherit spatial knowledge and reliability estimates from previous flights and from teammates, turning prior physical experience into future behavioral bias. The navigation strategy dynamically adapts trajectory points through this reward mechanism. Optimal outcomes reinforce decision weights $w^{L'_j}_{L_i}$, while suboptimal ones are penalized, refining the weight tensor $\omega _{L_i}$ through reinforcement learning. This process is physically analogous to “learning who to trust” in the field: nodes that repeatedly produce safe and efficient guidance gain more influence over future motion decisions of their peers.

With the understanding of how navigation points are defined, it is possible to roll back to image mapping to understand how a monocular vision of a Tello UAV was programmed to generate 3D points locally, relating the trajectory points to these. Basically, as UAVs fly, they generate local 3D world points in 2D images and share these points through the network, so it’s possible to use feature matching (Feature extraction and matching between image frames is implemented using OpenCV ORB and Brute-Force Matching libraries^33,34^.) techniques on those points to recognize corresponding world coordinates and apply epipolar geometry solutions and triangulation to both recreate 3D geometry and align trajectories. Physically, this means that different UAVs can mutually confirm the position of structural elements (e.g., walls, obstacles, doorways) and align their trajectories to a shared map, even if they observed those elements at different times. To ARCog-NET, this comes naturally because if a higher decision weight is found at the weight tensor, automatically it is related to the better trajectory coverages in the knowledge base found at that moment, and this also will indicate that the framework already has stored points of that region to serve as abase for 3D reconstruction and trajectory refinement. Therefore, high-weight guidance is not only “trusted” abstractly but is grounded in previously mapped, geometrically consistent regions of the physical environment.

**Fig. 3:**
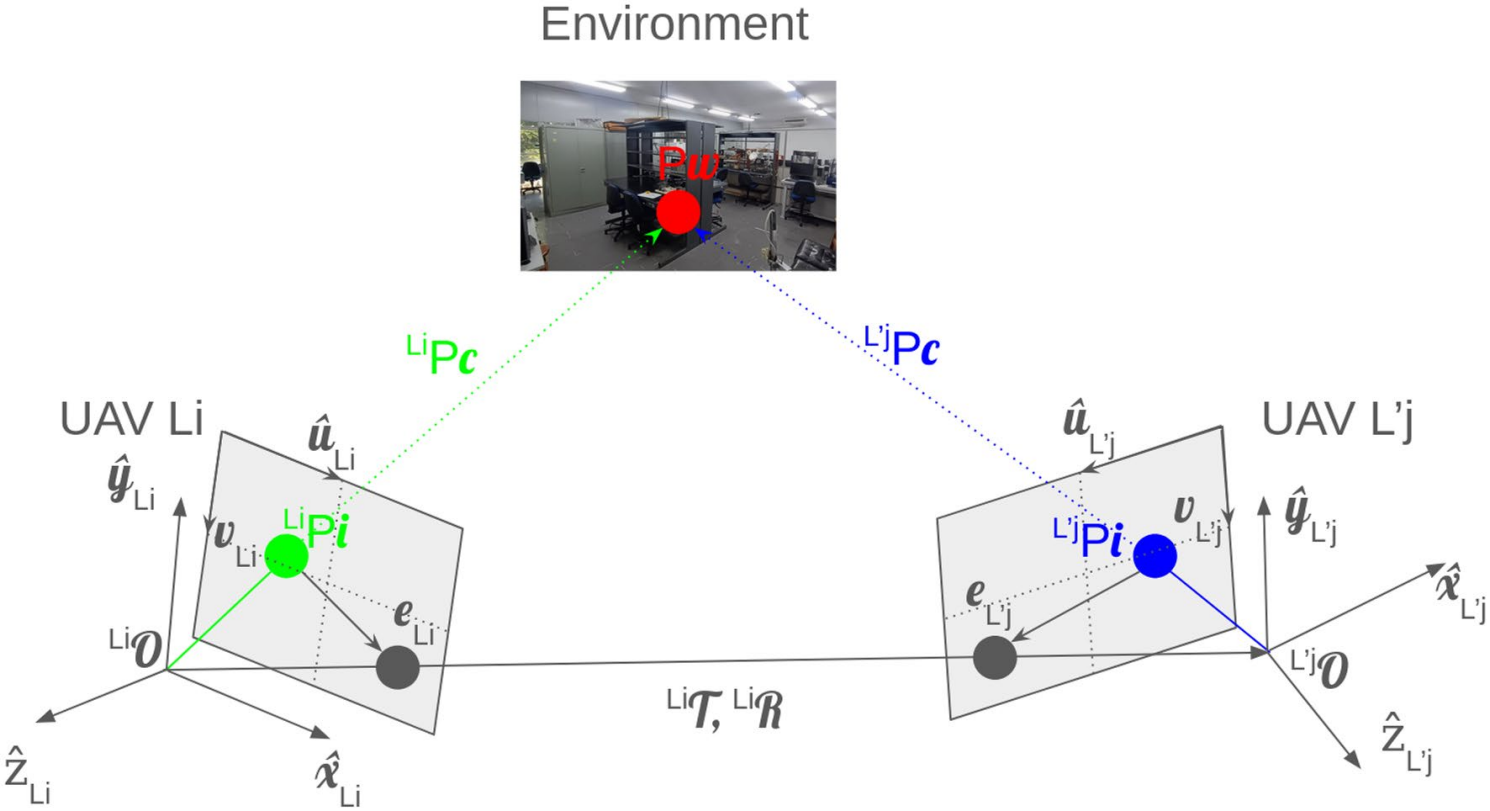
Epipolar geometry from point matching.

For instance, if a UAV $L_i$ encounters an obstacle (avoidance flight event) and has to recalculate its trajectory, it can search for a good decision weight in a similar task, indicating an optimal coverage solution previously registered by UAV $L'_j$. Concretely, this means UAV $L_i$ can immediately reuse a physically validated evasive maneuver that another UAV $L'_j$ already executed safely under comparable spatial constraints, rather than exploring a risky new avoidance path. This also indicates that, in $L'_j$ memory of image frames and points, some features that UAV $L_i$ is “seeing” can be matched, as illustrated in Figure 3. In physical terms, visual feature matching here confirms that both UAVs are in fact observing the same real structure in the environment (e.g., the same wall edge or doorway), enabling consistent multi-agent localization and collaborative SLAM.

To extract the exact global $P_w$ point from different images captured by different UAVs, the following system must be solved:

10 $$\begin{aligned} \begin{aligned} {\left\{ \begin{array}{ll} ^{L_i}P_c = ^{L_i}\mathscr {R} \, ^{L'_j}P_c + ^{L_i}\mathscr {T} & \quad (E1)\\ ^{L_i}P_c^\top E \, ^{L'_j}P_c = 0 & \quad (E2)\\ E = ^{L_i}\mathscr {R} \, ^{L_i}\mathscr {T} & \quad (E3)\\ u_{L_i} = ^{L_i}f_x \cdot \frac{^{L_i}X_i}{^{L_i}Z_c} + ^{L_i}O_x & \quad (E4)\\ v_{L_i} = ^{L_i}f_y \cdot \frac{^{L_i}Y_i}{^{L_i}Z_c} + ^{L_i}O_y & \quad (E5)\\ (^{L_i}\mathscr {K}^{-1})^\top F \, ^{L'_j}\mathscr {K} = E & \quad (E6)\\ ^{L_i}P_c^\top F \, ^{L'_j}X_i = 0 & \quad (E7) \end{array}\right. } \end{aligned} \end{aligned}$$

Physically, the system in (10) describes how two different UAVs observing the same real-world feature from different poses can agree on that feature’s 3D position in a shared reference frame. Equation (E1) expresses a rigid-body transformation between coordinate frames, where $^{L_i}\mathscr {R}$ and $^{L_i}\mathscr {T}$ represent the measured orientation (roll, pitch, yaw) and translation (in meters) of UAV $L_i$ with respect to UAV $L'_j$. This maps the 3D coordinates observed by UAV $L'_j$ into the camera frame of UAV $L_i$, enforcing geometric consistency between agents.

Equation (E2) is the epipolar constraint in 3D form: it encodes the fact that the two viewing rays from the cameras of UAV $L_i$ and UAV $L'_j$ toward the same physical point must lie on a common epipolar plane. This plane is described by the essential matrix $E$, which is defined in (E3) from the relative pose of UAV $L_i$. Operationally, this expresses the physical requirement that a landmark seen by two UAVs must be located along the intersection of their lines of sight.

Equations (E4) and (E5) describe the ideal pinhole projection model for UAV $L_i$’s camera. The pixel coordinates $(u_{L_i}, v_{L_i})$ are obtained from the true 3D location $(^{L_i}X_i, ^{L_i}Y_i, ^{L_i}Z_c)$ of that point relative to the UAV camera. The focal lengths $^{L_i}f_x$ and $^{L_i}f_y$ and the optical center offsets $^{L_i}O_x$ and $^{L_i}O_y$ are physical calibration parameters of the onboard camera (in pixels), meaning they directly encode how distances in meters project to displacements on the image sensor.

Equation (E6) links the fundamental matrix $F$ and the essential matrix $E$ through each UAV’s intrinsic calibration $\mathscr {K}$. This establishes how the purely geometric relationship between cameras in 3D ($E$) becomes a constraint on pixel correspondences ($F$). Equation (E7) is the classic 2D epipolar constraint: it enforces that, for a true physical point seen by both UAVs, the pixel location in one image must lie on the epipolar line induced by the corresponding pixel in the other image. In practice, this filters out false matches and ensures that only geometrically plausible point correspondences are accepted.

where $(E1)$ is the 3D to 3D transformation, $(E2)$ is the 3D epipolar constraint, $(E3)$ represent the essential matrix $E$, $(E4)$ and $(E5)$ are equations for perspective projections in $x$ and $y$ directions on the images of UAV $L_i$, respectively, being $^{L_i}f_x$ and $^{L_i}f_y$ the focal lengths on same directions. Finally, $(E6)$ represents the relation between the fundamental matrix $F$ and the essential matrix and $(E7)$ is the 2D epipolar constraint. Altogether, these equations formalize how two UAVs with known poses and calibrated cameras can triangulate the absolute 3D coordinates $P_w$ of a shared scene feature. This is the mathematical basis that allows collaborative mapping and consistent multi-agent SLAM in ARCog-NET, because it ties pixel measurements back to metric positions in the shared indoor environment.

In a subset of $n$ point correspondences (obtained solving equations $E1$ and $E2$), the fundamental matrices can be found solving:

11 $$\begin{aligned} \left[ {\begin{array}{ccccccccc} u_{1,L_i}u_{1,L'_j} & v_{1,L'_j}u_{1,L_i} & u_{1,L_i} & u_{1,L'_j}v_{1,L_i} & v_{1,L_i} & u_{1,L'_j} & v_{1,L'_j} & 1\\ u_{2,L_i}u_{2,L'_j} & v_{2,L'_j}u_{2,L_i} & u_{2,L_i} & u_{2,L'_j}v_{2,L_i} & v_{2,L_i} & u_{2,L'_j} & v_{2,L'_j} & 1\\ u_{3,L_i}u_{3,L'_j} & v_{3,L'_j}u_{3,L_i} & u_{3,L_i} & u_{3,L'_j}v_{3,L_i} & v_{3,L_i} & u_{3,L'_j} & v_{3,L'_j} & 1\\ \vdots & \vdots & \vdots & \vdots & \vdots & \vdots & \vdots & \vdots \\ u_{n,L_i}u_{n,L'_j} & v_{n,L'_j}u_{n,L_i} & u_{n,L_i} & u_{n,L'_j}v_{n,L_i} & v_{n,L_i} & u_{n,L'_j} & v_{n,L'_j} & 1 \end{array} } \right] \left[ {\begin{array}{c} F_{1} \\ F_{2} \\ F_{3} \\ \vdots \\ F_{n} \end{array}}\right] = 0 \end{aligned}$$

Equation (11) shows how the fundamental matrix $F$ is estimated from multiple matched pixel pairs $(u_{k,L_i}, v_{k,L_i})$ and $(u_{k,L'_j}, v_{k,L'_j})$ observed by UAV $L_i$ and UAV $L'_j$. Each correspondence comes from a real physical feature (e.g., a corner, edge, or textured surface) that both UAVs see from different viewpoints. Solving this linear system yields $F$, which encodes the epipolar geometry between the two UAVs’ cameras and therefore captures their relative pose.

**Fig. 4:**
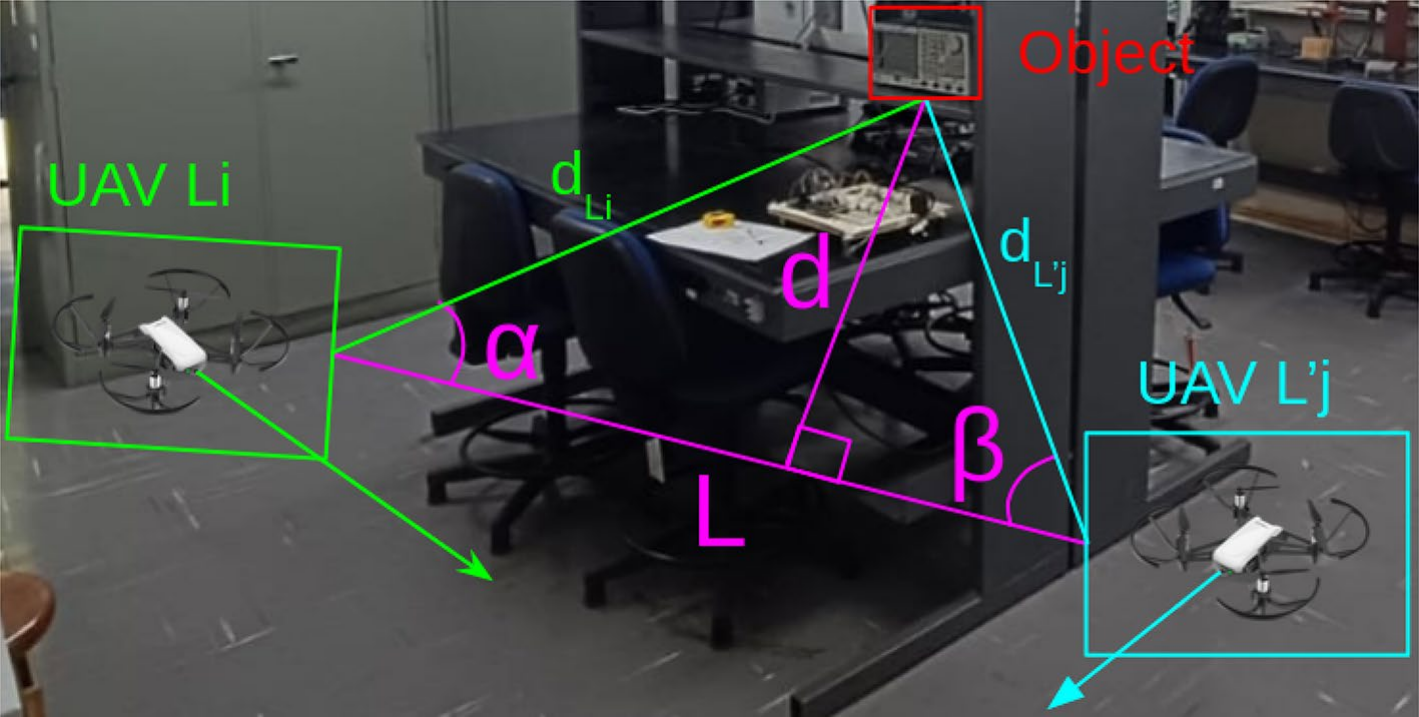
Triangulation to form the 3D point cloud from matching image points and camera parameters.

By using OpenCV’s libraries to solve the system and find the fundamental matrices^35^, it’s possible to find all other parameters through the system of Equations (10) and therefore obtain the UAV (camera) position related to the matching points from the history of image frames. In practice, this means that after estimating $F$, ARCog-NET can infer where each UAV was located and oriented when it observed a point, and can then triangulate the 3D position of that point in the shared global frame. Knowing the UAV’s position and its motion through trajectories, it is possible to estimate the spatial position of the matching points in the local 3D world coordinate frame, as illustrated in Figure 4. This allows the swarm to build a consistent joint point cloud of the environment, aligned in metric space, which is essential for collaborative navigation, collision avoidance, and coordinated coverage in GPS-denied indoor missions.

Knowing all presented camera calibration parameters and matching points, suppose $(u_{L_i}, v_{L_i})$ be the coordinates of a point in the image frame of the UAV $L_i$ and $(u_{L'_j}, v_{L'_j})$ the corresponding point in the image frame of the UAV $L'_j$. The projection matrices for the two camera poses between the initial time $t_i$ and the next captured frame time $t+\tau$ are defined as $^{L_i}P_c = ^{L_i}\mathscr {K}[^{L_i}I \mid 0]$, $^{L'_j}P_c = ^{L'_j}\mathscr {K}[^{L'_j}\mathscr {R} \mid ^{L'_j}\mathscr {T}]$. The relationship between a 3D point $P_w$ and its 2D projection $P_i$ in an image is given by the camera projection model $P_i = P_cP_w$. Thus, the projections in both images are $^{L_i}P_i = ^{L_i}P_c^{L_i}P_w$, $^{L'_j}P_i = ^{L'_j}P_c^{L'_j}P_w$. Since the cross product of a vector with itself is zero, and a valid projection must lie along the projected ray, it’s possible to derive the following constraints $^{L_i}P_i \times (^{L_i}P_c^{L_i}P_w) = 0$, $^{L'_j}P_i \times (^{L'_j}P_c^{L'_j}P_w) = 0$.

These cross-product constraints ensure that the point $P_w$ lies along the correct epipolar lines in both images. Expanding these equations yields a system of linear constraints, typically assembled into a matrix *A*, such that $A P_w = 0$. The solution to this homogeneous system is found using the Singular Value Decomposition (SVD) of *A*, which provides the least-squares estimate of the 3D point $P_w$ in homogeneous coordinates.

Notice that this is a local point, as it is formed by a UAV *i* in layer *L*. Usually, edge agents will fly more, collecting more points and sending them to their fog coordinators. The fog coordinators usually process the mathematics to group the 3D points of their cluster, generating the cluster 3D point cloud. Finally, the server receives all data from the network and can generate the global 3D reconstruction, feeding back the network with the global map, which is aligned to the trajectories of each component, allowing the system to fly while mapping the environment.

This combination allows ARCog-NET to improve trajectories as the mission unfolds and prevent collisions or other situations that will menace UAV flight. When events demand trajectory re-planning, ARCog-NET invokes its internal navigation algorithm, which leverages past experience and global mapping to optimize future choices. Previously computed trajectories are revisited, and if suitable, reused, which reduces the computational burden. The algorithm uses real-time metrics to update weights and decision matrices.

In the context of ARCog-NET, cognition refers to the algorithmic process through which each agent perceives its environment, interprets contextual data, recalls prior experiences from the distributed knowledge base, and adapts its decision policy accordingly. This cognitive behavior emerges from three interdependent mechanisms: (i) adaptive decision-weight reinforcement, where agents continuously update the influence of neighboring nodes based on temporal-difference learning (Eq. (14)); (ii) knowledge reuse, in which past coverage performance and guidance quality are recorded in the trajectory history matrix $H_{L_i}(t)$ (Eq. (8)) and its associated decision weight matrix $W_{L_i}(t)$ (Eq. (9)), allowing an agent to reuse previously validated solutions instead of replanning from scratch; and (iii) multi-layer inference, in which an agent at layer *L* (edge, fog, or cloud) can request or provide trajectory adaptations based on physically grounded motion models and coverage rewards, using the kinematic update laws in Eqs. (5)–(6) together with the coverage-driven performance signal $C^{\text {total}}_{L_i}$ in Eq. (7) to bias future actions toward higher-yield exploration under current mission constraints. Therefore, cognition in ARCog-NET is a functional, quantifiable process that integrates perception, memory, and adaptive decision-making rather than a descriptive abstraction.

To explicitly define the adaptive update process of the decision weights, the reinforcement learning component in ARCog-NET models the UAV’s decision-making as a discrete-time optimization problem in which each agent *i* in layer *L* selects an action $a^{L_i}_t$ (e.g., trajectory adjustment or coordination choice) given its current state $s^{L_i}_t$, producing a reward $r^{L_i}_t$. The cumulative objective for each UAV is to maximize the expected discounted return:

12 $$\begin{aligned} J^{L_i} = \mathbb {E}\left[ \sum _{t=0}^{T} \gamma ^t , r^{L_i}_t \right] , \end{aligned}$$

where $\gamma \in [0,1]$ is the temporal discount factor. The reward function is defined as a weighted sum of mission-relevant performance indicators:

13 $$\begin{aligned} r^{L_i}t = \alpha _{C , C^{\text {total}}_{L_i}}(t) + \alpha _{E , E_{L_i}}(t) + \alpha _{R , R_{L_i}}(t) + \alpha _{L , L_{L_i}}(t), \end{aligned}$$

where $C^{\text {total}}{L_i}(t)$ denotes the trajectory coverage defined in Equation (7), $E{L_i}(t)$ the normalized energy consumption, $R_{L_i}(t)$ the collision-risk indicator derived from inter-agent proximity, and $L_{L_i}(t)$ the decision latency of the node in milliseconds, all normalized in [0, 1]. The coefficients $\alpha _C$, $\alpha _E$, $\alpha _R$, and $\alpha _L$ are tunable hyperparameters that balance exploration quality, safety, and resource usage. At every decision step, the weight matrix $W_{L_i}(t)$ is updated according to the temporal-difference rule:

14 $$\begin{aligned} w^{L'j}_{L_i}(t+1) = w^{L'j}_{L_i}(t) + \eta \cdot \delta ^{L_i}_t, \end{aligned}$$

where $\eta$ is the learning rate and $\delta ^{L_i}_t$ is the temporal-difference error computed as:

15 $$\begin{aligned} \delta ^{L_i}_t = r^{L_i}_t + \gamma \cdot \max \{{a'} Q^{L_i}(s_{t+1}, a') - Q^{L_i}(s_t, a_t)\}, \end{aligned}$$

with $Q^{L_i}(s_t, a_t)$ representing the estimated value of performing action $a_t$ in state $s_t$. Each decision weight $w^{L'j}_{L_i}$ therefore represents the learned quality of adopting trajectory recommendations from node *j* at time *t*, gradually converging as the agent accumulates successful outcomes. The learned weights are periodically normalized:

16 $$\begin{aligned} w^{L'j}_{L_i}(t) \leftarrow \frac{w^{L'j}_{L_i}(t)}{\sum _{k=1}^{N} w^{L'k}_{L_i}(t)}, \end{aligned}$$

ensuring that influence among peer or superior nodes remains bounded and interpretable. To ensure reproducibility and isolate the contribution of each cognitive component prior to field trials, a simulation-based ablation is performed using digital UAV models and ARCog-NET topology within a indoor scenario (see the third scenario from Ramos et al. 2025^12^), comparing three reduced configurations against the full ARCog-NET: (i) without decision-weight reinforcement (no *w*-update), where agents follow Equation (7) but do not adapt weights; (ii) without knowledge reuse (frozen $\kappa _{L_i}$), forcing purely local decisions and disabling cross-layer sharing; and (iii) without Edge–Fog–Cloud coordination, removing fog intermediaries so all agents communicate directly with the cloud. For each configuration, the ATE, RPE, trajectory coverage, knowledge reuse rate, decision latency, and failure recovery rate are recorded. This protocol quantifies the marginal effect of each component and demonstrates whether reinforcement-based weighting yields measurable gains in accuracy and robustness before real-world experimentation. Finally, the detailed navigation loop is presented in Algorithm 1.

**Algorithm 1:**
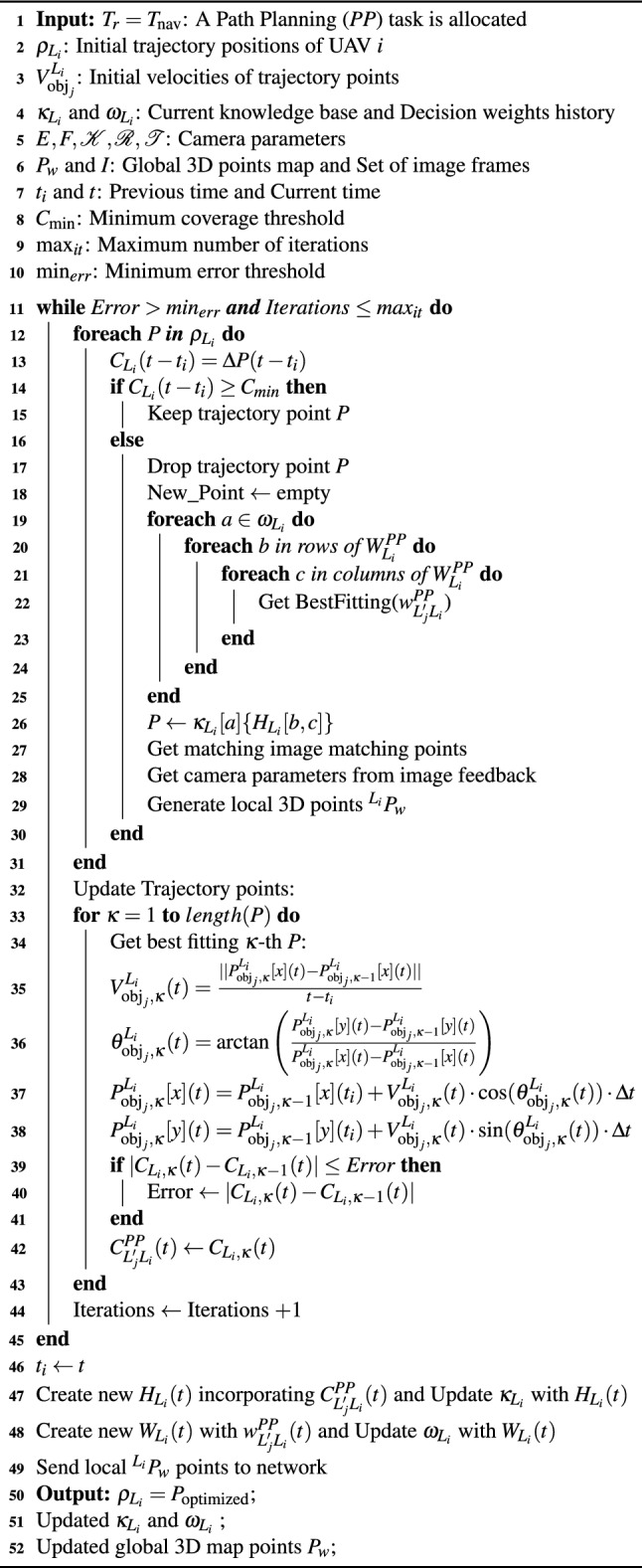
ARCog-NET navigation event trajectory path planning.

Upon task execution, the agent assesses its success through updated coverage. If improved, the results are reinforced and recorded. If no solution proves suitable, the UAV escalates the request to its fog node. Fog nodes replicate this mechanism with requests from edge agents and intra-fog events. Effective solutions are stored, weights are updated, and feedback loops reinforce beneficial actions. The cloud layer follows the same cognitive loop, processing trajectory requests with global situational awareness. It stores, validates, and transmits high-level optimized decisions back to the requester, closing the loop. This architecture empowers autonomous path planning under uncertainty, ensuring high responsiveness, formation integrity, and efficient map building in SLAM-enabled ARCog-NET deployments.

To evaluate the adaptive capability of the proposed architecture when exposed to dynamic disturbances, a metric named the Adaptive Response Index (ARI) was developed. This index quantifies how effectively the system reacts to and recovers from events described before. The ARI varies within the interval [0, 1], where values close to one indicate an excellent response characterized by fast reaction, limited degradation, and stable recovery, while values near zero indicate poor adaptability.

For each disturbance event starting at time $t_0$, the evaluation considers several measurable components that describe the dynamic response of the system. The first component is the detection delay, which represents the time required for the controller to recognize that a disturbance has occurred or that any monitored metric has exceeded its tolerance band. It is defined as

17 $$\begin{aligned} \tau _{\textrm{det}} = \min \{\, t \ge t_0 : |m_k(t) - \mu _k|> \varepsilon _k \text { for any } k \,\} - t_0, \end{aligned}$$

where $m_k(t)$ is the observed value of metric *k*, $\mu _k$ is its steady-state reference, and $\varepsilon _k$ is the acceptable tolerance around that value. Once a disturbance is detected, the magnitude of the degradation is assessed through the maximum normalized deviation across all monitored variables, expressed as

18 $$\begin{aligned} \Delta _{\max } = \max _{k} \, \max _{t \in [t_0, t_1]} \left( \frac{|m_k(t) - \mu _k|}{\varepsilon _k} \right) . \end{aligned}$$

After the disturbance, the system begins to stabilize, and the time required for it to return and remain within the nominal performance band is represented by the recovery time, defined as

19 $$\begin{aligned} \tau _{\textrm{rec}} = \min \left\{ t \ge t_0 : |m_k(u) - \mu _k| \le \varepsilon _k,\, \forall k,\, \forall u \in [t, t + \theta ] \right\} - t_0, \end{aligned}$$

where $\theta$ corresponds to the minimum period of sustained stability required to consider the recovery complete. The intensity and duration of the degradation are jointly represented by the degradation area, given by

20 $$\begin{aligned} \textrm{ID} = \sum _{k} \int _{t_0}^{t_1} \left[ \frac{|m_k(t) - \mu _k| - \varepsilon _k}{\varepsilon _k} \right] _+ dt, \end{aligned}$$

where $[x]_+ = \max (x, 0)$ denotes the positive part operator. This formulation aggregates both the amplitude and persistence of performance deviations across all monitored quantities. In addition to these behavioral indicators, a reconfiguration cost term is introduced to account for the system’s internal effort to adapt during the disturbance. This cost is defined as

21 $$\begin{aligned} C_{\textrm{rec}} = c_R N_{\textrm{roles}} + c_E E_{\textrm{sup}} + c_Q Q_{\textrm{fog}}, \end{aligned}$$

where $N_{\textrm{roles}}$ represents the number of edge/fog role transitions, $E_{\textrm{sup}}$ is the additional energy consumption in watt-hours, and $Q_{\textrm{fog}}$ is the average backlog increase observed in the fog layer during adaptation. The coefficients $c_R$, $c_E$, and $c_Q$ assign relative importance to each cost component depending on mission constraints. In practice, the end of observation $t_1$ can be defined as the end of the experimental window or as a fixed period after the disturbance, typically 60 s, and the stability threshold $\theta$ is often between 2 and 5 s. Each component is normalized by its respective reference limit to produce dimensionless quantities, according to

22 $$\begin{aligned} \hat{\tau }_{\textrm{det}} = \frac{\tau _{\textrm{det}}}{\tau _{\max }}, \qquad \hat{\tau }_{\textrm{rec}} = \frac{\tau _{\textrm{rec}}}{T_{\max }}, \qquad \widehat{\textrm{ID}} = \frac{\textrm{ID}}{\textrm{ID}_{\max }}, \qquad \hat{C}_{\textrm{rec}} = \frac{C_{\textrm{rec}}}{C_{\max }}. \end{aligned}$$

The overall adaptive response for a given event *e* is then computed by combining these normalized terms into a single scalar indicator, as shown in

23 $$\begin{aligned} \textrm{ARI}_e = 1 - \left( w_1 \hat{\tau }_{\textrm{det}} + w_2 \hat{\tau }_{\textrm{rec}} + w_3 \widehat{\textrm{ID}} + w_4 \hat{C}_{\textrm{rec}} \right) , \qquad \textrm{ARI}_e \in [0,1], \end{aligned}$$

where $w_1 + w_2 + w_3 + w_4 = 1$. The weights $w_i$ define the relative influence of detection speed, recovery duration, degradation severity, and adaptation cost, respectively, according to the priorities of the evaluated mission. A higher ARI indicates a system capable of quickly identifying perturbations, limiting the magnitude of performance losses, and recovering stability with minimal internal overhead. When multiple disturbance events occur within an experiment, an aggregated value can be obtained by computing the mean index across all events, expressed as

24 $$\begin{aligned} \overline{\textrm{ARI}} = \frac{1}{E} \sum _{e=1}^{E} \textrm{ARI}_e, \end{aligned}$$

and complementary indicators such as the 5th, 50th, and 95th percentiles ($\textrm{ARI}_{p5}$, $\textrm{ARI}_{p50}$, $\textrm{ARI}_{p95}$) can be used to evaluate consistency across repeated trials. In the experimental setup involving six DJI Ryze Tello UAVs, tolerance bands included a maximum deviation of $0.05\,\textrm{m}$ for absolute trajectory error (ATE), $40\,\textrm{ms}$ for network latency, and $0.10\,\textrm{m}$ for formation error. The selected weights were $w_1 = 0.2$, $w_2 = 0.4$, $w_3 = 0.3$, and $w_4 = 0.1$, emphasizing recovery stability over instantaneous response. This metric provides a systematic means of comparing adaptive performance among different ARCog-NET configurations and quantifying, in a consolidated manner, the system’s robustness under real dynamic indoor conditions.

To validate the model, an experiment was performed using 6 DJI Ryze Tello UAVs connected in the ARCog-NET framework to map the Control and Automation Laboratory (LACEA) of the Federal Center for Technological Education Celso Suckow da Fonseca (CEFET-RJ). The environment is composed of 5 environments, being the experimentation area, the workstations area, and 3 personal rooms. The UAV’s initial information was only the $(x, y)$ coordinate of the deploy point of each UAV in relation to the lowest left corner of the laboratory as the origin of the world coordinate frame. To navigate and map the laboratory, the swarm used only the cognitive architecture for navigation and communication provided by ARCog-NET and the new implementation of the image SLAM, updating position based on Equation (6), aligning trajectory points to world point cloud $P_w$ and updating it on time, velocity and heading angle with Equation (5).

**Fig. 5:**
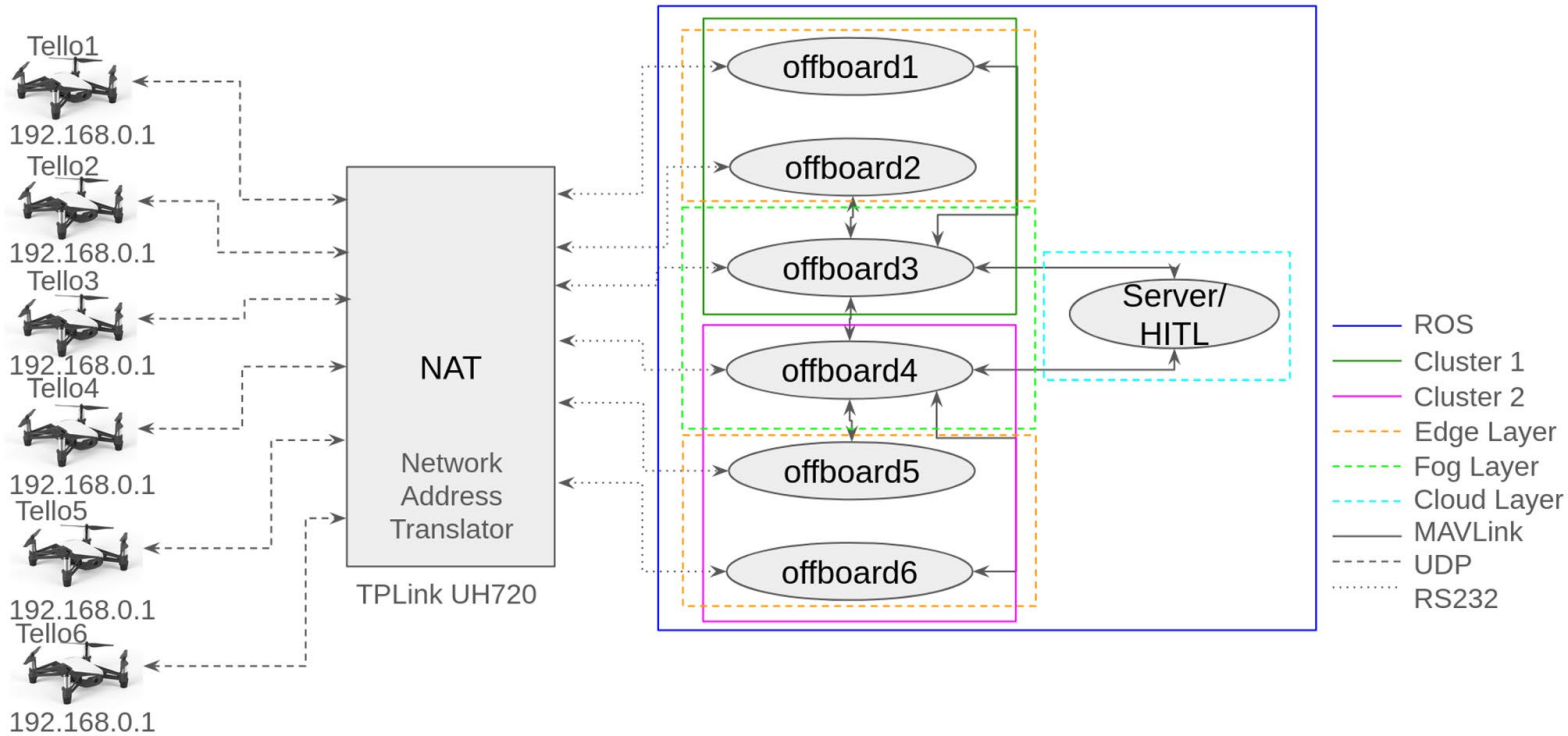
Laboratory test setup with 6 DJI Ryze Tello connected to ARCog-NET.

The Tellos were connected to a NAT (Network Address Translator), each using a dedicated Wi-Fi card, which directed each UAV’s UDP communication to a respective offboard ROS node running on a laptop connected to the NAT. Using the Tello Standard Development Kit (SDK)^36^ to read basic data from the UAVs, which were captured by the ROS offboard nodes and processed within ARCog-NET, sending back flight commands to the UAVs also through the SDK. The ROS offboard nodes communicate with each other within the edge-fog-cloud structure of ARCog-NET through MAVLINK^21^ protocol over MAVROS^19^ package. The 6 offboards are divided into 2 groups of 3 Tellos. Each group had one fog coordinator and two edge agents. During the mission, the edge and fog roles switched between offboards (or Tello) depending on the UAV status and mission tasks. Only the coordinator (fog) communicates with the server (cloud), also running as a single ROS node. The experiment setup is presented in Figure 5.

To evaluate the performance of the ARCog-NET framework integrated with Monocular Visual SLAM in a multi-UAV indoor mapping task, several quantitative and qualitative metrics were employed. SLAM performance was assessed through Absolute Trajectory Error (ATE), Relative Pose Error (RPE), and reprojection error, ensuring trajectory consistency and map fidelity. The quality of the 3D reconstruction was also evaluated in terms of completeness and point cloud density. For the cognitive and networked aspects, metrics included trajectory coverage score, decision weight convergence, knowledge reuse rate, and replanning frequency, reflecting the system’s learning and adaptability. Network performance was analyzed through decision latency across Edge-Fog-Cloud layers, bandwidth usage, and computational load distribution. Additionally, robustness was measured by the rate of failure recovery and the frequency of UAV role-switching between edge and fog nodes. Finally, the proposed ARI was used for measuring adaptability response to events during the mission. These metrics collectively enabled a comprehensive assessment of the system’s mapping accuracy, decision intelligence, and communication efficiency. The next session will explore the obtained results and discussions originating from the laboratory tests.

## Results and discussion

**Fig. 6:**
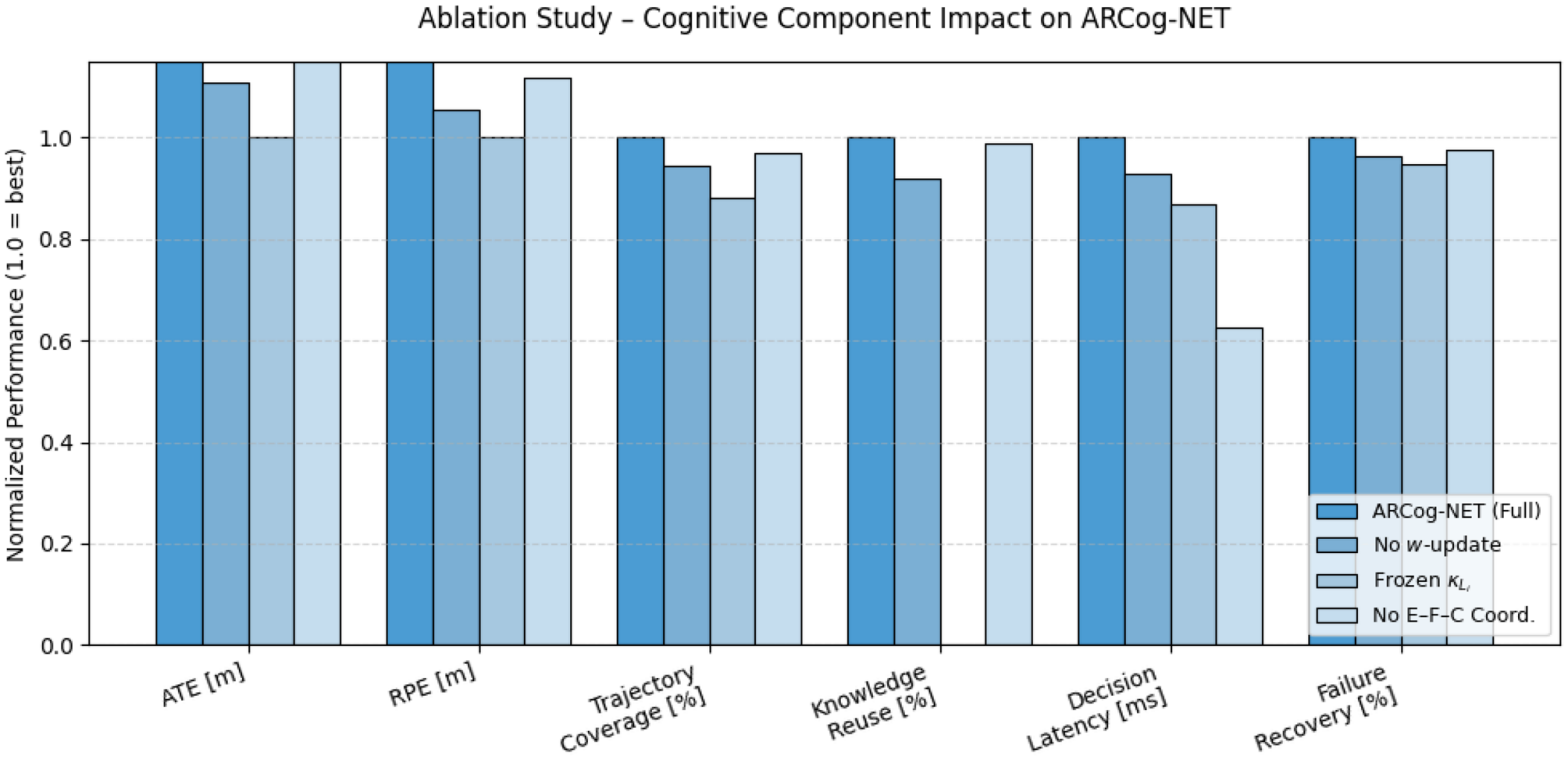
Ablation study of the simulated ARCog-NET cognitive architecture.

The simulation results of indoor environment mission to perform the ablation studies regarding the impact of cognitive parameters to ARCog-NET’s performance are presented in Figure 6.

The ablation analysis in Figure 6 demonstrates that the full ARCog-NET configuration outperforms all reduced variants across nearly every metric, confirming the synergistic contribution of its cognitive modules. The absence of decision-weight reinforcement (*w*-update) leads to moderate degradation in coverage and recovery, while freezing knowledge reuse ($\kappa _{L_i}$) causes the most significant drop in overall adaptability, indicating the centrality of experience sharing in cooperative learning. The removal of Edge–Fog–Cloud coordination mainly affects decision latency, validating the role of hierarchical communication in maintaining low network response times. Altogether, these results confirm that reinforcement learning, knowledge reuse, and multi-layer coordination jointly enhance the accuracy, resilience, and efficiency of ARCog-NET in dynamic swarm operations.

To provide a ground-truth reference for SLAM accuracy evaluation, all external and internal walls of the laboratory were precisely measured prior to the experiment, and the initial take-off positions of the six UAVs were marked relative to the lower-left corner of the environment reference frame. In this coordinate system, the global positive *x*-axis extends to the right and the positive *y*-axis extends forward, perpendicularly to *x*-axis, from that origin. This setup allowed each UAV’s estimated trajectory to be compared with its physically measured ground-truth displacement, enabling direct calculation of ATE and RPE values throughout the mission. The experiment was executed as described in the previous section, was recorded on video^37^ and analyzed on site.

**Fig. 7:**
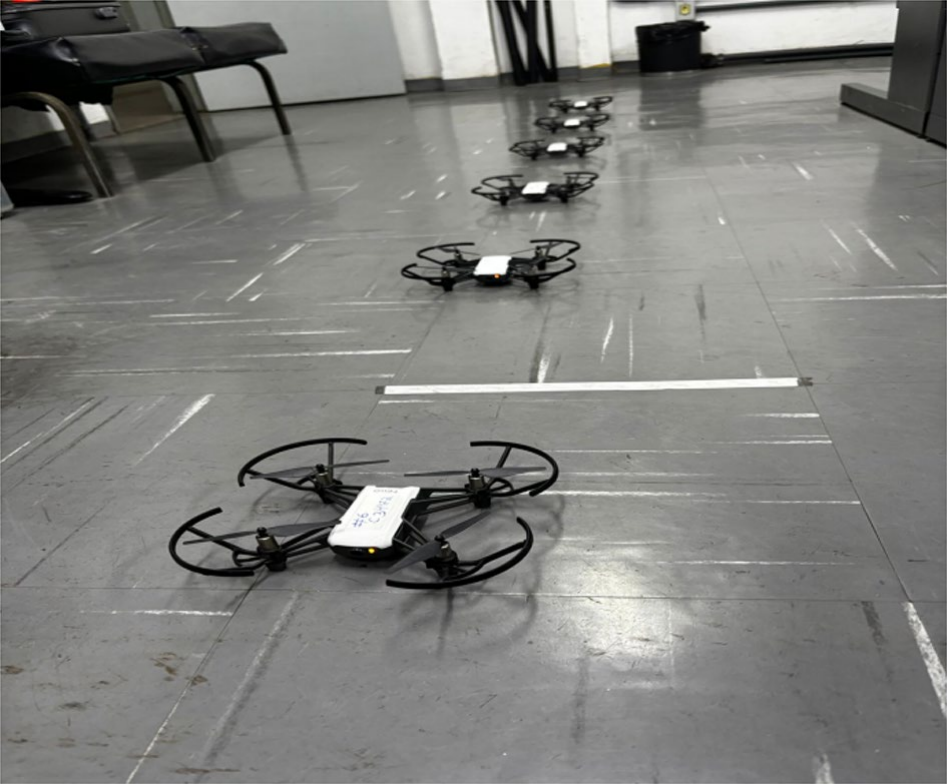
Swarm picture before the take off.

The UAV took flight from the initial positions, from points $\{(1.5, 1.5, 0)$, (2.0, 1.5, 0), (2.5, 1.5, 0), (3.0, 1.5, 0), (3.5, 1.5, 0), $(4.1, 1.5, 0)\}$, defined with reference to the lower-left corner of the laboratory and meticulously measured, for Tello $\#1$ to Tello $\#6$. The UAVs $\#3$ and $\#4$ were initially configured as fog layer nodes, with the first being the fog coordinator of the edge group composed of UAVs $\#1$ and $\#2$ and the second being the fog coordinator of the edge group composed of UAVs $\#5$ and $\#6$. The clusters changed configuration during mission (*e.g.*, an agent became a coordinator and vice-versa), but never changing elements between clusters and never having more than one coordinator per cluster. Figure 7 is a picture of the swarm taken before the test starts, and an image extracted from RQT Graph that illustrates the active ROS nodes and topics at the deployment is available on ARCog-NET’s GitHub repository^38^.

**Fig. 8:**
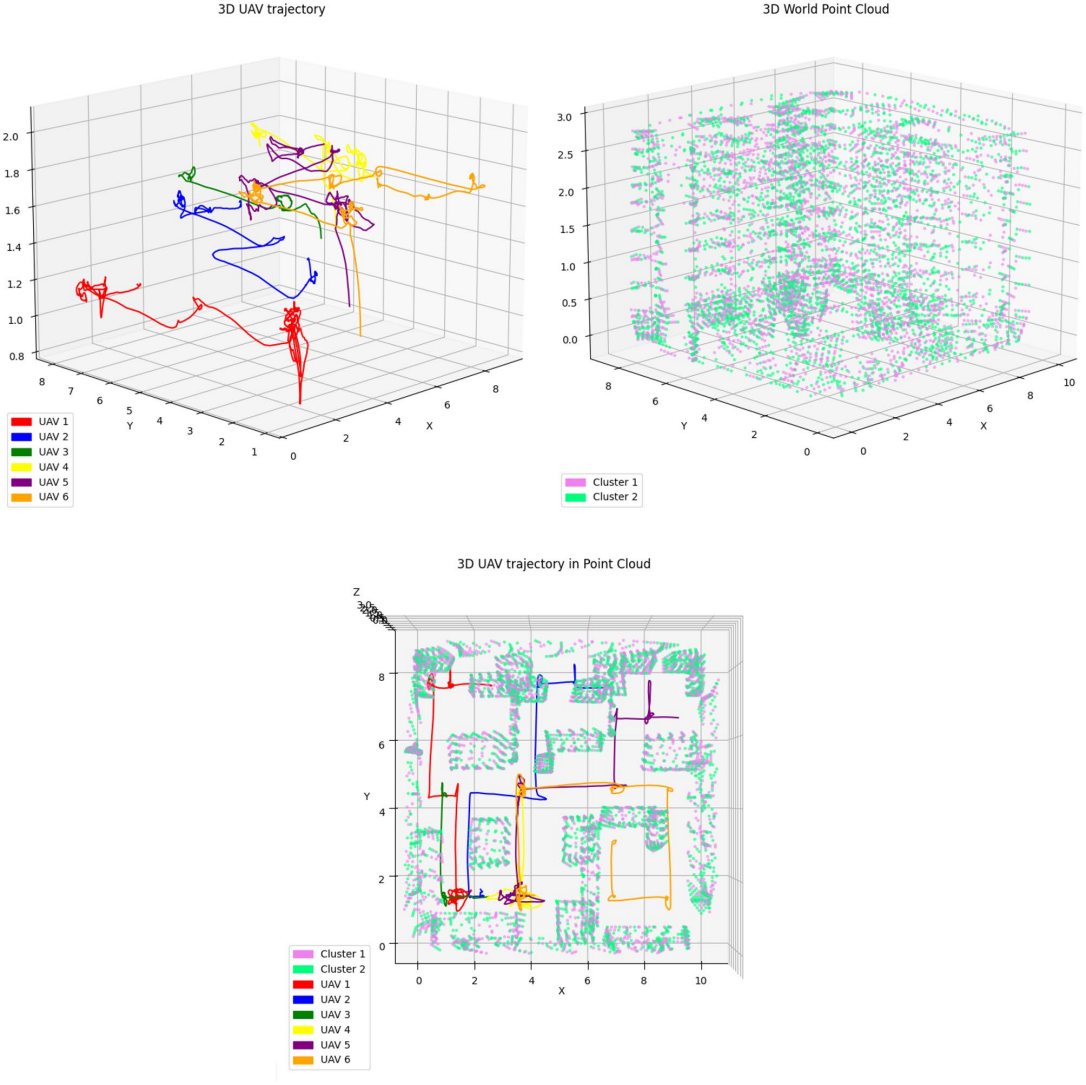
3D reconstruction of the environment through point clouds (high resolution images available at the ARCog-NET’s GitHub^39^).

The UAVs were able to travel and map the entire laboratory environment, forming the 3D point cloud of the whole environment by sharing only the image points, their yaw angles, and forward traveled distances, as presented in the video^37^. The swarm took 3 minutes and 15 seconds to scan all environment, totaling 93.98 m$\phantom{0}^2$ in area, gathering 7006 points (Notice that those are the matched points used to cloud layer to reconstruct the environment. During the mission, lots of features (points) locally gathered by each UAV as it executed the mission were collected, but weren’t used in the global map.). The scanned environment represented by the 3D point cloud alongside the flight trajectories executed by the Tello UAVs is presented in Figure 8.

All datasets and algorithms are linked in the Data Availability section at the end of the paper. The numerical results, using the analysis settings described in Methods and Modelling, are reported in Table 2.

**Table 2:** Benchmarked Performance Metrics of ARCog-NET Integrated with Monocular Visual SLAM.

Metric	Friess ^24^	Xu ^25^	Zhong ^26^	Aldossary ^27^	Hu ^28^	Zhang ^32^	Zhang ^31^	Liu ^29^	Liu ^30^	ARCog-NET (this work)
**SLAM Accuracy**										
Absolute Trajectory Error (ATE) [m]	0.12	0.25	0.18	–	–	0.27	0.24	0.034	0.032	$0.21 \pm 0.05$
Relative Pose Error (RPE) [m]	–	0.17	–	–	–	–	0.16	0.018	0.021	$0.14 \pm 0.03$
Reprojection Error [px]	–	–	–	–	–	–	–	1.4	1.2	$1.27 \pm 0.29$
**3D Reconstruction**										
Completeness [%]	–	–	–	–	–	–	85.1	87.3	88.5	$87.6 \pm 3.2$
Point Cloud Density [points/m$\phantom{0}^2$]	–	–	–	–	–	–	68	72	74	$75 \pm 5$
**Cognitive Performance**										
Trajectory Coverage Score [%]	–	–	–	–	–	90.5	92.3	93.1	94.8	$91.2 \pm 2.7$
Decision Weight Convergence [iter]	–	–	–	–	–	–	6.1	5.8	5.5	$5.4 \pm 0.9$
Knowledge Reuse Rate [%]	–	–	–	–	–	–	–	61.2	63.8	$64.3 \pm 4.1$
Replanning Frequency [#/mission]	–	–	–	–	–	11.0	9.8	10.6	9.9	$10.2 \pm 1.1$
**Network Metrics**										
Decision Latency (E–F–C) [ms]	–	–	–	$\sim$55	$\sim$48	–	–	46.8	21.9	$42.5 \pm 8.3$
Bandwidth Usage [MB/min]	–	–	–	15.4	13.8	–	–	2.81	3.25	$12.7 \pm 1.9$
Computational Load (E/F/C) [%]	–	–	–	40/44/16	38/46/16	–	–	42/45/13	44/41/15	35/42/23
**Robustness**										
Failure Recovery Rate [%]	–	–	–	92.1	93.4	90.2	91.5	95.3	94.7	$94.8 \pm 1.7$
Role Switching Frequency [#/mission]	–	–	–	1.7	1.3	–	–	1.4	1.5	$1.5 \pm 0.6$
Average ARI [0–1]	–	–	–	–	–	–	–	0.86	0.87	$0.88 \pm 0.04$

The results presented in Table 2 demonstrate the comprehensive performance of the ARCog-NET architecture when integrated with Monocular Visual SLAM in the context of collaborative indoor mapping with UAV swarms. Regarding SLAM accuracy, the obtained Absolute Trajectory Error (ATE) of $0.21 \pm 0.05$ meters and Relative Pose Error (RPE) of $0.14 \pm 0.03$ meters confirm that the system maintains high precision in tracking the estimated path of the UAVs throughout the mission. This is particularly relevant given the limited sensing and processing capabilities of lightweight platforms such as Tello drones. Additionally, the reprojection error of $1.27 \pm 0.29$ pixels demonstrates good consistency between image observations and the projected 3D structure, indicating accurate estimation of camera pose and map points.

The benchmarking results also shown in Table 2 highlight that **ARCog-NET** achieves a balanced and comprehensive performance across all evaluated dimensions. In terms of SLAM accuracy, its ATE of 0.21 m and RPE of 0.14 m are competitive with the best distributed systems, approaching the precision of Friess et al. (2023)^24^ while surpassing both the decentralized D$\phantom{0}^2$SLAM^25^ and the LiDAR-based DCL-SLAM^26^. Although previous works such as Aldossary et al. (2024)^27^ and Hu et al. (2025)^28^ emphasized fog–cloud optimization, they did not address cognitive adaptation at the swarm level, resulting in higher latency and less balanced computational loads. More recent single-agent frameworks by Zhang et al. (2024)^32^ and Zhang et al. (2025)^31^ advanced path planning and exploration through reinforcement learning and visibility-graph optimization, achieving strong trajectory adaptation and local robustness but remaining limited to non-distributed implementations.

ARCog-NET, in its turn, integrates these learning concepts within a hierarchical Edge–Fog–Cloud structure, combining adaptive cognitive planning, multi-layer coordination, and collective knowledge reuse. Consequently, it sustains high 3D reconstruction completeness (87.6%), efficient communication (12.7 MB/min), and robust operation with a 94.8% recovery rate and an average ARI of 0.88. These results demonstrate that ARCog-NET not only matches but extends state-of-the-art frameworks by bridging individual learning and distributed cognition into a unified, resilient swarm architecture. When compared with recent edge-assisted and centralized collaborative SLAM systems, ARCog-NET demonstrates a broader integration of cognitive reasoning and hierarchical communication. Liu et al^29^. achieved outstanding precision in visual–inertial odometry (ATE $=0.034$ m) and efficient data compression with low bandwidth use (2.8 MB/min), but their edge framework lacks multi-layer coordination or adaptive cognitive weighting. Similarly, the centralized CPL-SLAM^30^ reached even lower latency (21.9 ms) and high accuracy (ATE $=0.032$ m), yet depends on a fixed global fusion node, limiting scalability and robustness under degraded communication. ARCog-NET, though slightly less precise in pure odometry, surpasses both in adaptability and resilience by dynamically balancing edge, fog, and cloud layers, achieving higher recovery rates (94.8%) and sustained multi-agent autonomy through reinforcement-driven decision weighting and distributed knowledge reuse.

In terms of computational performance, the results reported by Liu *et al.*^29,30^ demonstrate that both the Edge-Assisted Multi-Robot Visual-Inertial SLAM and CPL-SLAM frameworks operate with considerably lower computational burden compared to ARCog-NET. Specifically, Liu *et al.*^29^ achieve real-time distributed SLAM performance with an average processing rate of approximately 30 Hz and a total CPU utilization below 45%, enabled by efficient feature compression and edge-assisted optimization. Likewise, Liu *et al.*^30^ report a centralized collaborative SLAM achieving nearly 32 Hz, with about 40–45% CPU usage on the front-end and 55–57% on the back-end for feature fusion and global optimization. In contrast, the proposed ARCog-NET introduces additional computational overhead due to its cognitive reasoning layer, reinforcement-based trajectory adaptation, and multi-layer coordination processes, resulting in a lower average processing rate of around 18 Hz and a higher CPU utilization near 80%. This comparison highlights the trade-off between efficiency-oriented SLAM architectures and cognitively enhanced swarm frameworks such as ARCog-NET, which favor autonomy, resilience, and cooperative intelligence over raw throughput.

**Fig. 9:**
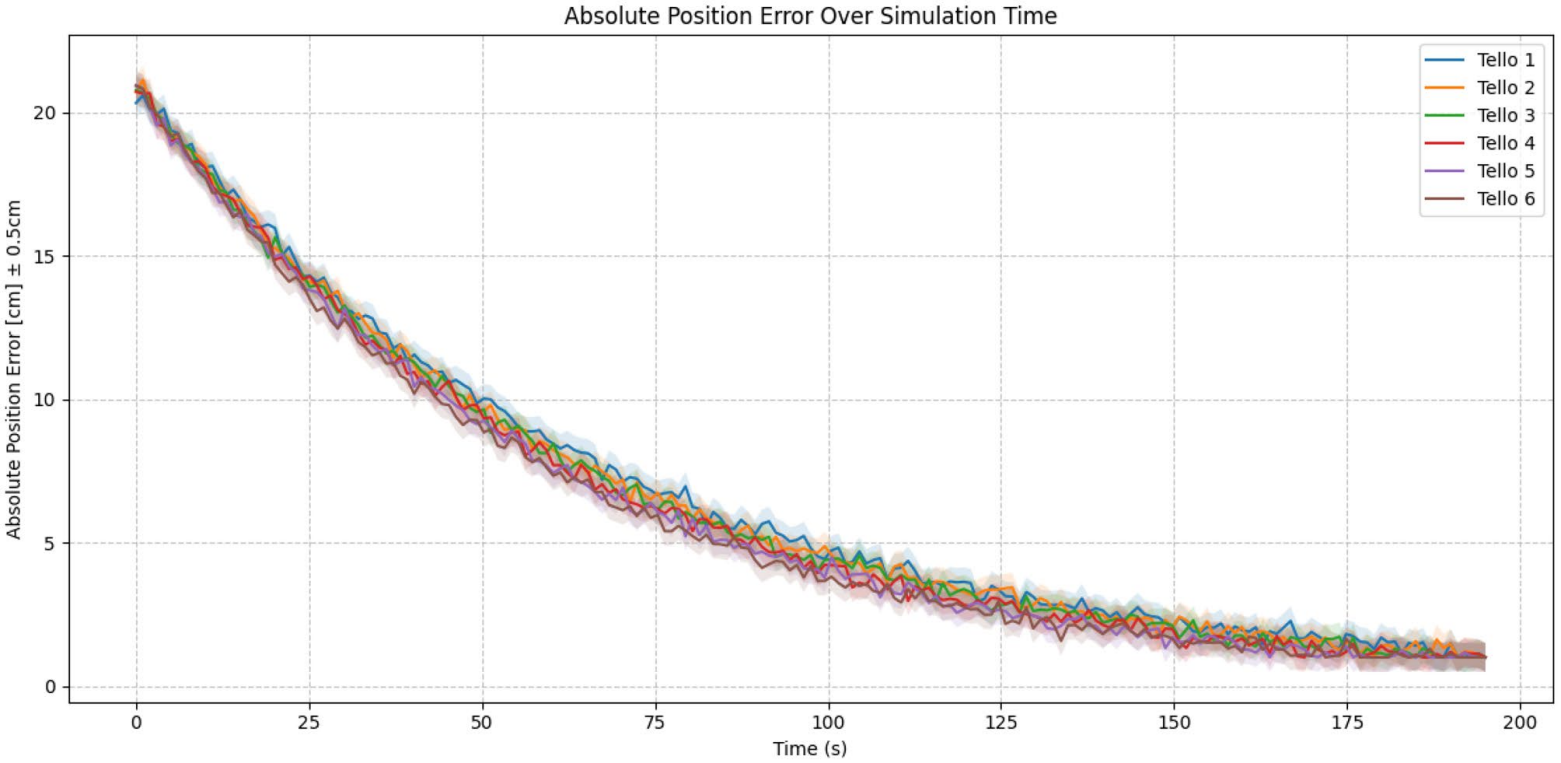
Absolute position error over time.

Regarding the positioning improvements achieved over time, given the reinforcement learning process, the graph in Figure 9 illustrates a progressive reduction in absolute position error across all six Tello drones during the 195-second experiment, confirming the effectiveness of the ARCog-NET framework integrated with monocular SLAM. Initially, errors exceed 20 cm, reflecting limited spatial awareness at startup, but steadily decrease to under 2 cm as the swarm explores, maps, and reinforces positional accuracy through shared cognition and coordinated adaptation. The consistent decay curves and tight uncertainty bounds ($\pm 0.5$ cm) across all agents indicate both high reliability and convergence, validating the system’s ability to enhance localization through distributed learning and real-time inter-agent cooperation.

The divergence and subsequent reconvergence observed in Figure 9 reflect the adaptive and cooperative behavior of the UAVs during the SLAM learning phase. At the beginning of the experiment, each Tello operates with partially independent trajectories and distinct calibration data, leading to absolute position errors that diverge as local odometry and perception inaccuracies accumulate. As the mission progresses, ARCog-NET’s knowledge-sharing mechanism, particularly between edge agents and fog coordinators, enables the exchange of visual landmarks and corrected trajectories, driving the reconvergence of individual errors. The slight offset among UAVs, with some maintaining consistently lower or higher errors, results from differences in flight paths, visual exposure (lighting, occlusions, and field of view), and role transitions within the swarm. Overall, the initial divergence followed by convergence demonstrates the cooperative self-alignment process of the system: as the shared map consolidates and the collective estimation refines, position error stabilizes around 1–2 cm after approximately 180 s of flight.

The 3D reconstruction performance reinforces these findings, with the completeness of the map reaching $87.6 \pm 3.2\%$, which means most of the environment was effectively reconstructed through collaborative mapping. The point cloud density of $75 \pm 5$ points per square meter shows a sufficient level of detail in the reconstructed model, which is critical for applications requiring spatial awareness, such as inspection, navigation, or semantic labeling. These results indicate that the shared mapping process using ORB-SLAM observations across multiple UAVs was effective, and the fusion mechanism preserved a coherent and dense global representation.

From a cognitive standpoint, ARCog-NET’s capabilities are demonstrated by a trajectory coverage score of $91.2 \pm 2.7\%$, confirming that the swarm was capable of efficiently exploring the designated area. The decision weight convergence occurring within $5.4 \pm 0.9$ iterations illustrates the rapid learning and stabilization of the decision-making process, which contributes to minimizing unnecessary replanning or inconsistencies during flight. The knowledge reuse rate of $64.3 \pm 4.1\%$ highlights that UAVs frequently reuse previously learned strategies, reducing redundant computations and improving response time. The replanning frequency of $10.2 \pm 1.1$ actions per mission reflects an adaptive behavior without excessive instability, enabling UAVs to dynamically adjust their behavior in response to environmental variations or neighbor behavior.

The network-related metrics confirm that the Edge-Fog-Cloud architecture is suitable for real-time operation. The measured decision latency of $42.5 \pm 8.3$ ms represents the average time between a UAV’s request and the corresponding control response, including both transmission and computation components. It arises from Wi-Fi communication between Edge and Fog, and MAVROS communication delay to cloud nodes (2–10 m range, signal above $-60$ dBm), and from simulated onboard reinforcement-learning processing on offboard nodes and cloud servers. This value, therefore, reflects the combined effects of data transmission, processing, and coordination within the distributed hierarchy under near-line-of-sight indoor conditions. Bandwidth usage remained moderate at $12.7 \pm 1.9$ MB per minute, benefiting from data filtering and compression mechanisms embedded in the architecture. Computational load distribution among Edge (35%), Fog (42%), and Cloud (23%) layers was well-balanced, ensuring that Edge UAVs retained sufficient autonomy while still benefiting from collective planning and optimization processes.

Robustness was another strong point of the system. The failure recovery rate of $94.8 \pm 1.7\%$ indicates that the swarm could handle agent failures or data inconsistencies with minimal performance degradation. Furthermore, the role-switching frequency of $1.5 \pm 0.6$ per mission reflects the capacity of UAVs to dynamically transition between edge and fog roles when required by the context or mission state, reinforcing resilience and decentralization.

**Fig. 10:**
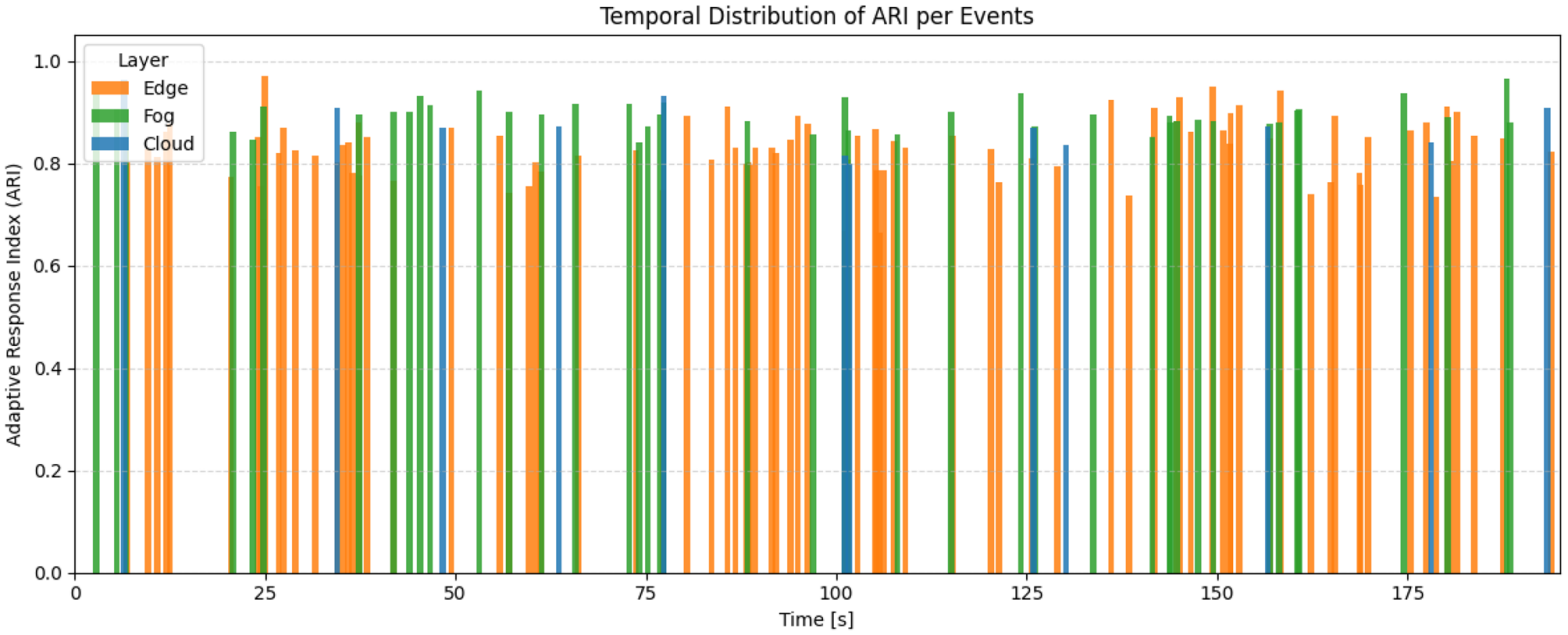
ARI results per detected event and layer during experiment.

In Figure 10, higher ARI values near 1.0 indicate fast detection and recovery with minimal degradation, while lower values correspond to delayed or unstable responses. Edge events occur more frequently and exhibit slightly lower but more dynamic ARI levels, consistent with their role in immediate local reactions and limited processing capability. Fog events appear less numerous yet more stable, maintaining ARI values close to 0.9 due to higher processing precision and supervisory coordination. Cloud events are sparse and represent high-level interventions triggered by global decisions or network commands; their ARI values remain intermediate, reflecting slower but robust responses that stabilize the swarm after large-scale disturbances. Notice that some events are responded to by more than one layer, indicating that this was an event escalated from a lower layer to a higher one, or a command passed from a higher layer to a lower one.

**Fig. 11:**
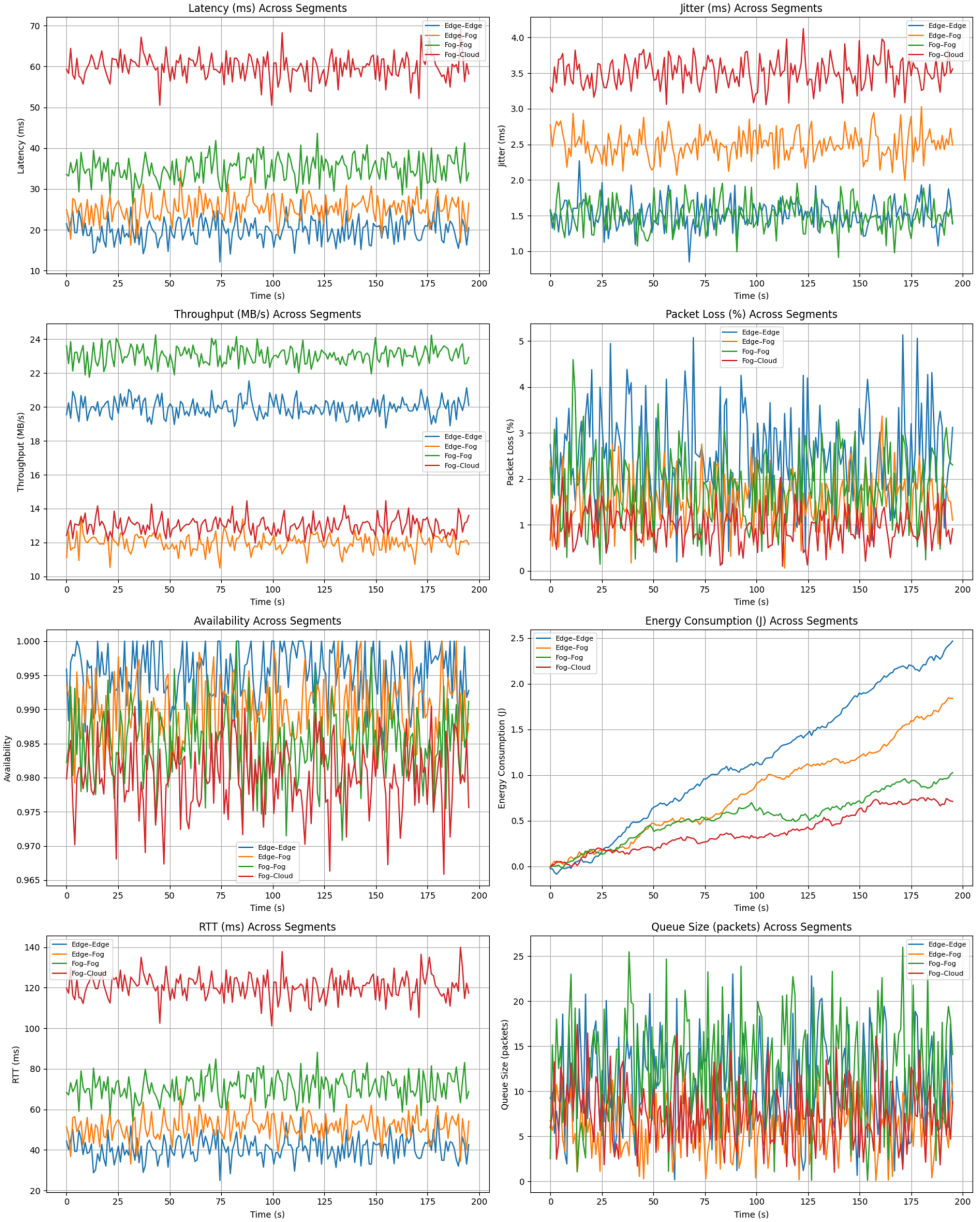
Network segments parameters.

Collectively, the temporal distribution of ARI reveals that the ARCog-NET architecture maintains high adaptability and resilience under dynamic indoor conditions, with the Edge–Fog–Cloud layers complementing each other in time and precision. Specific internal and external layer segments parameters, such as latency, jitter, RTT (Round Trip Time), throughput, packet loss, energy consumption, queue size, and availability, were also evaluated and presented in Figure 11.

The latency graph demonstrates a hierarchical structure in communication delay, with Edge-to-Edge connections showing the lowest latency, typically within the range of 15 to 25 milliseconds. This fast response is essential for decentralized control and local coordination. Edge-to-Fog communication presents slightly higher latency, between 25 and 35 milliseconds, indicating the cost of interfacing with intermediate nodes. Fog-to-Fog links vary between 35 and 45 milliseconds, while Fog-to-Cloud displays the highest latency, reaching values between 60 and 70 milliseconds. This validates the design principle of ARCog-NET in which high-latency decisions are deferred to the cloud layer, and latency-sensitive tasks remain within the edge and fog layers.

Jitter, which quantifies latency variability, follows a similar gradient. Edge-to-Edge and Fog-to-Fog maintain stable jitter levels around 1.5 to 2 milliseconds, ensuring predictable communication needed for formation and trajectory control. Edge-to-Fog shows moderate jitter around 2.5 milliseconds. Fog-to-Cloud again displays the highest jitter, often reaching 3.5 to 4 milliseconds. These results reinforce the notion that the ARCog-NET architecture successfully isolates temporal variability from time-critical decision paths.

Throughput is highest in Fog-to-Fog communications, exceeding 25 MB/s, likely due to the role of fog nodes in aggregating data from edge agents and disseminating coordination messages. Edge-to-Edge links also exhibit high throughput (approximately 22 MB/s), reflecting real-time exchange of navigation and SLAM data. Conversely, Edge-to-Fog and Fog-to-Cloud links handle reduced data rates, around 11 to 14 MB/s, which suggests effective data filtering and compression strategies prior to offloading, as intended by the architecture.

Packet loss varies across segments. While generally low, Edge-to-Edge and Edge-to-Fog links exhibit occasional bursts of loss above 5%, likely caused by environmental interference or wireless congestion. Fog-to-Fog and Fog-to-Cloud links remain more stable, typically below 2.5%, suggesting robustness of higher-level communication aided by error correction or retransmission mechanisms. The system’s resilience to data loss may be credited to cognitive decision smoothing and redundancy embedded in the ARCog-NET structure.

Availability remains consistently high across all segments. Edge-to-Edge links operate near full availability, supporting high-frequency swarm coordination. Edge-to-Fog and Fog-to-Fog maintain availability between 97.5% and 99%, while Fog-to-Cloud drops occasionally below 97.5%. These slight fluctuations at the cloud layer emphasize the importance of limiting dependence on remote servers for core swarm functions.

Energy consumption analysis reveals that Edge-to-Edge communications are the most energy intensive, reaching over 2.5 Joules by the end of the experiment. This is expected given the high data exchange rate and autonomous computation required at the edge level. Edge-to-Fog communications are slightly less costly, followed by Fog-to-Fog and finally Fog-to-Cloud, which incurs the lowest cumulative energy consumption (approximately 1.3 Joules). These results confirm that decentralization trades energy for speed and autonomy.

RTT (Round Trip Time) measurements mirror the latency trends. Fog-to-Cloud again incurs the highest RTT, often above 120 milliseconds. Fog-to-Fog and Edge-to-Fog are moderately delayed (70–90 milliseconds), while Edge-to-Edge remains the most responsive path with RTT values consistently under 65 milliseconds. These measurements further support the tiered design of the system, with real-time processing confined to lower-latency layers.

Queue size fluctuations across segments are comparable, with short bursts reaching up to 15–20 packets. Despite transient peaks, all segments stabilize quickly, indicating effective buffer and scheduling policies. Edge-Fog and Fog-Fog exhibit slightly more variability, which may stem from their roles in coordinating between layers and handling aggregated traffic. Overall, queues are well-managed, preventing systemic bottlenecks even under active data flows.

The stability and scalability of the proposed control are consistent with prior simulations of ARCog-NET conducted in an industrial indoor environment, where up to ten UAVs performed cooperative missions under dynamic, obstacle-rich and communication-limited conditions^12^. In that study, navigation accuracy remained high, with average MAE (Mean Absolute Error) below 0.05 *m* and RMSE (Root Mean Squared Error) under 0.12 *m*, while time shifts between planned and executed trajectories (from −77 *ms* to +63 *ms*) reflected adaptive role transitions between edge and fog layers without compromising control quality. The present six-UAV experiment exhibited similar dynamic behavior, confirming that the hierarchical and distributed Edge–Fog–Cloud model maintains control stability and communication resilience under comparable indoor conditions. However, scalability tests previously performed with up to 1000 UAVs demonstrated that, although ARCog-NET preserves coordination and decision efficiency as swarm size grows, performance improvements gradually saturate beyond a few hundred agents, as communication overhead and computational demand increase faster than the resulting gains in trajectory planning or task allocation. This confirms that the framework scales efficiently within practical operational ranges, but excessive swarm expansion yields diminishing returns due to the natural saturation of the distributed cognitive model.

In summary, the architecture demonstrates a strong match between the communication infrastructure and its cognitive, layered design. Edge-level communications offer low latency and high availability suitable for real-time swarm operations, while fog and cloud layers support coordination and supervision without impeding responsiveness. The system effectively balances throughput, energy efficiency, and robustness, validating its application in real-world, indoor multi-UAV missions.

The ARCog-NET framework presented excellent performance in terms of navigation accuracy, environment reconstruction, cognitive learning, communication efficiency, and operational robustness. These characteristics make it a strong candidate for deployment in real-world scenarios where UAVs operate in dynamic and resource-constrained environments. The next section will further analyze comparative results with existing state-of-the-art frameworks in order to contextualize the significance of these findings.

## Conclusions

This study introduced and experimentally validated the ARCog-NET framework, a distributed cognitive architecture designed to support the development of intelligent and scalable multi-robot swarms. The framework was conceived to be generic and flexible, enabling adaptation across diverse domains, including mapping, inspection, search and rescue, and environmental monitoring. In this work, ARCog-NET was integrated with Monocular Visual SLAM and deployed in a real-world indoor mapping experiment using a swarm of six UAVs. The experiment demonstrated how ARCog-NET’s cognitive structure-based on decision weight learning, historical knowledge reuse, and layered coordination-contributed to more efficient and robust swarm behavior.

Key contributions of this work include the successful demonstration of distributed SLAM-based reconstruction using shared visual data, the application of reinforcement learning mechanisms to improve trajectory planning and formation control, and the implementation of cooperative knowledge fusion across Edge, Fog, and Cloud layers. The framework enabled real-time adaptation to dynamic conditions, efficient bandwidth use, and reduced human intervention through progressive autonomy. The experiment validated ARCog-NET’s capacity to handle coordination, mapping, and decision-making tasks in a synchronized and resilient manner, reinforcing its role as a practical enabler of intelligent swarm robotics.

## Data Availability

All experiment data and ARCog-NET experiment and simulation codes (from the cited published works) are available in main Author’s GitHub repository^12^.

## Declarations

### Patents

The main ARCog-NET Python files developed packages have been patented as a computer software under Brazilian jurisdiction, receiving the certificate $BR512024001222-2$ emitted by the National Institute of Industrial Property of the Federal Brazilian Government at the issue 2781 of the weekly Patent Magazine in April 24, 2024^40^.
